# Improving Agri-environmental Schemes: Suggestions from Farmers and Nature Managers in a Central European Region

**DOI:** 10.1007/s00267-023-01922-w

**Published:** 2023-12-18

**Authors:** Manuela Zindler, Maria Haensel, Ute Fricke, Thomas M. Schmitt, Cynthia Tobisch, Thomas Koellner

**Affiliations:** 1 Professorship of Ecological Services, Bayreuth Center of Ecology and Environmental Research (BayCEER), University of Bayreuth, Universitätsstraße 30, 95447 Bayreuth, Germany; 2 Department of Animal Ecology and Tropical Biology, Julius-Maximilians-University, Am Hubland, 97074 Würzburg, Germany; 3 Institute of Ecology and Landscape, Weihenstephan-Triesdorf University of Applied Sciences, Am Hofgarten 4, 85354 Freising, Germany

**Keywords:** Agri-environmental climate measures, Nature protection, Farmer survey, Stakeholder involvement, Cluster analysis, Content analysis

## Abstract

Agri-environmental schemes (AES) are important policy instruments within the Common Agricultural Policy (CAP) of the European Union for environmental protection. Due to the voluntary nature of AES, their attractiveness to farmers and stakeholders involved in nature management and protection (nature managers) is essential for high participation levels. This study aims to assess farmers’ and nature managers’ ideas to improve agri-environmental schemes. We analyzed suggestions of 825 farmers and 118 nature managers for improvements of AES collected in a large-scale survey in Bavaria, Germany. A content analysis was applied to categorize and compare suggestions by farmers (differentiated into two groups through a cluster analysis) and nature managers. The results reveal that stakeholders were highly willing to share ideas and made detailed suggestions for improvements and individual measures. They were aware of the importance of protecting nature and promoting biodiversity in agricultural landscapes and acknowledged the necessity of (financial) support programs. Farmers placed more emphasis on the practicability and profitability of measures on arable land, while nature managers tended to propose policy-related ideas focusing on nature protection, biodiversity, and specific species. Among farmers, suggestions differed with farm characteristics such as the operation mode (full-time, part-time). These findings can support the design of future AES, accounting for different background situations and thereby increasing acceptability. This includes considering perspectives from different stakeholder groups and creating regionally adapted programs with varying levels of flexibility and practicability.

## Introduction

Increased agricultural intensification associated with large amounts of fertilizers, pesticides, and monocultures threatens biodiversity and negatively affects the environment and ecosystem service provisioning (Tscharntke et al. 2005; IPBES 2019). Ecosystem services and biodiversity are essential to provide a good quality of life. Given the need to provide food for an increasing population, strategies to reduce the impacts of agricultural practices have to be sought (IPBES 2019). The importance of strengthening environmentally friendly agriculture has also been acknowledged by changes in the agricultural policy system and related subsidies. In the case of the Common Agricultural Policy of the European Union (CAP), agri-environmental schemes (AES) have been supported since 1992 (Mennig and Sauer 2019). Farmers who participate in these voluntary schemes receive financial compensation for the loss incurred by lower productivity that may occur due to the utilization of less intensive and more environmentally friendly farming practices (Batáry et al. 2015). Between 2014 and 2020, around 24.4% (99.59 billion €) of the CAP budget was allocated for rural development. From this share for rural development, at least 30% had to be spent on environmental and climate-related objectives, including AES (Massot 2021).

The large CAP budget offers great potential to improve the environmental state in agriculturally dominated landscapes, though ongoing discussions about allocating this money most efficiently remain (Heyl et al. 2021). Integrating perspectives from various stakeholders, including farmers and personnel involved in nature management and protection (nature managers), represents a valuable way to exploit this potential and improve schemes (Toderi et al. 2017). In our study, nature managers represent a variety of actors working or volunteering in organizations and agencies related to nature protection and management of greenspaces (see Section “Survey of farmers and nature managers”). The involvement of stakeholders can further improve the attractiveness of schemes. Given the voluntary nature of AES, this is essential to increase participation and achieve environmental targets (Espinosa‐Goded et al. 2010). A study by Prager and Freese (2009) showed that including farmers’ practical knowledge in AES could increase the attractiveness and acceptability of schemes.

Given that farmers’ incomes depend on agricultural production, it can be expected that they would formulate ideas emphasizing production-integrated measures, addressing feasibility and practicalities. On the other hand, professionals in nature protection might focus more on nature conservation than agricultural productivity. Thus, incorporating the perspectives of nature managers and farmers could support the creation of attractive schemes that aim to minimize trade-offs between additional work, productivity loss, and environmental benefits. Additionally, flexibility and adequate compensation are important to increase attractiveness for stakeholder groups (Lastra-Bravo et al. 2015).

Many studies have addressed the design and acceptability of AES (Uthes and Matzdorf 2013) by examining factors for participation (Defrancesco et al. 2008; Lastra-Bravo et al. 2015; Buschmann and Röder 2019), the ecological or cost-effectiveness of schemes (Ekroos et al. 2014; Batáry et al. 2015; Ansell et al. 2016), and the connection between AES and ecosystem services (Früh-Müller et al. 2018). Some studies discuss specific measures. For example, Sattler and Nagel (2010) investigated the risks, practicability, and costs of certain AES. Christensen et al. (2011) as well as Mante and Gerowitt (2009) analyzed single measures, including pesticide-free buffer zones and field margins, respectively. Many studies evaluated factors for the attractiveness of AES based on choice models, with a predefined set of answer options, which gives little room for comments from the survey respondents (Buschmann and Röder 2019; Defrancesco et al. 2008; Ruto and Garrod 2009).

It is important to note that the opinions of non-farmer stakeholders involved in AES are underrepresented in the literature. Some rare examples address the attitudes of both farmers and policy administrators (Schulze and Matzdorf 2023), perspectives of farm advisors (Hejnowicz et al. 2016), the role of landscape management associations as bridging institutions that support collaboration and coordination (Prager 2015; Josefsson et al. 2017), as well as the impact of landscape management associations on the implementation of AES (Schomers et al. 2015). However, investigating the perspectives of these stakeholders is particularly important, given their role as implementing entities or advisors. Considering the intensity with which the agricultural sector shapes landscapes, there is a need to engage multiple stakeholders, including practitioners in nature protection, in the design of AES (Prager and Freese 2009).

Likewise, too little attention has been paid to the impact of various farmer characteristics on their perspectives regarding AES. Previous studies have been primarily based on single variables, such as the farm system (conventional, organic) or farmer education status. However, multiple other economic, social, and farm characteristics, including factors like the operation mode (full-time, part-time) and the farm size, have been identified as factors playing a role in the acceptability of AES. Differences can also be expected due to varying levels of environmental awareness (Lastra-Bravo et al. 2015). Investigating the preferences of distinct groups can help design more targeted measures, potentially increasing participation rates (Barreiro-Hurlé et al. 2010).

Our study fills a gap by systematically asking farmers and nature managers (stakeholders involved in nature management and protection) about their ideas for improving funding schemes for protecting nature and landscapes in agricultural areas. By analyzing data from a large-scale survey in Bavaria, Germany, we aim to identify recommendations and required adaptations for agri-environmental schemes by addressing the following research questions:

1. What suggestions do farmers and nature managers have to improve AES in Bavaria, and how do these differ between farmers and nature managers and among farmers?
2. How does the frequency of suggested AES compare to current AES participation rates in Bavaria?

## Material and Methods

### Study Area

This study was conducted in Bavaria, Germany, in 2020. The major land use types in this area are arable land (46%) and forest (35%), which account for more than 2/3 of the land area (70,542 km²) (StMELF 2021). As arable land dominates Bavarian landscapes, AES are an important instrument for ensuring environmental protection. Three AES types exist in Bavaria: KULAP, VNP, and LNPR. The oldest and largest program is the Bavarian Cultural Landscape Program (KULAP, *Kulturlandschaftsprogramm*), introduced in 1988. For a detailed description of target areas and implementation of the KULAP, see Mennig and Sauer (2019). A second, smaller AES is the Nature Conservation Program (VNP, *Vertragsnaturschutzprogramm*), introduced in 1996. The VNP accounted for about 24% of the budget for KULAP and VNP between 2014 and 2022 and focuses on biotopes, protected areas, and the conservation of species, particularly meadow-breeding birds. VNP measures largely have specific requirements regarding area setting (e.g., located within protected areas) and need the approval of the lower nature conservation authority. Both of these AES types have a standard contract term of five years (LfL 2018). Every second farm business participated in at least one measure in 2020. In 2020, 105,000 farm businesses existed in Bavaria, 48% smaller than 20 ha and 6% larger than 100 ha (StMELF 2021). The programs are co-financed through the Rural Development Program by the European Union, Germany, and Bavaria (StMELF 2021). A complete list of measures can be found in the Supplementary Tables A1 and A2. A third small program through which Bavaria supported around 4000 measures in 2021 are the Guidelines for Landscape Conservation and Nature Parks (LNPR, *Landschaftspflege- und Naturparkrichtlinien*). These guidelines were introduced in 2014. They focus on nature and species protection and aim to support the Natura 2000 area and the Bavarian biotope network. Funding is granted by the state of Bavaria (StMUV 2023).

### Survey of Farmers and Nature Managers

We assessed farmers’ and nature managers’ perspectives on AES through a comprehensive, Bavarian-wide survey in 2020. We also collected data on socio-demographic and sectoral factors, such as farm management. Other parts of the survey focused on perceptions of ecosystem services (Thiemann et al. 2022), landscape elements (Küchen et al. 2023), and climate change (Landwehr et al. 2023). The mentioned studies include all four sampled societal actor groups (comprising foresters and citizens). For farmers, the online survey was partly supported by on-site sampling in agricultural offices. Nature managers (only online sampling) represent a variety of actors working or volunteering in organizations and agencies related to nature protection and management of greenspaces (Supplementary Table B1). For detailed information on the survey structure and sampling process, see Thiemann et al. (2022). In this study, we focused on the open-ended question: “What improvements to existing or new funding opportunities should there be for the protection of nature and landscapes in Bavaria?”. This question implicitly linked to AES for most respondents, as a choice experiment addressing AES was displayed to almost 90% of the participants prior to the question. Respondents could make up to three suggestions.

Our question of interest was answered by 825 farmers (of a total of 1738). Of those, 80% managed their own farm business, and 38% practiced farming full-time, matching Bavarian-wide statistics (StMELF 2021). After removing two outliers with large grassland areas (>12,600 hectares), our sample’s farm sizes and cropland and grassland shares matched the Bavarian statistics well. However, organic farming was overrepresented in our sample, with a share of 33% compared to 11% in the Bavarian average (ibid.). Overrepresentation was also true for farmers participating in AES (66% versus 54% for KULAP, 29% versus 20% for VNP) (ibid.). For nature managers, 118 (out of 207) answered the relevant question. Of these, 79% were professional, 38% were voluntary, and 18% were both professional and voluntary nature managers. Most participants worked in nature conservation areas (92%) or urban greenspaces (36%), with 30% working in both area types. Nature managers were predominately active in grasslands, wetlands, and water bodies. The majority (60%) did not receive subsidies for the managed areas.

### Differentiation between Farmers

To differentiate farmers’ response patterns, we created groups based on similar characteristics. We applied a factor analysis on mixed data, followed by hierarchical clustering as applied by Beltrán-Alcrudo et al. (2018) and Shukla et al. (2019) to construct farmer typologies. All analyses were performed in R (version 4.0.3) using the FactoMineR package (Lê et al. 2008). We validated the optimal number of clusters with the NbClust package (Charrad et al. 2014). Cluster variables consisted primarily of socio-demographic data, farm characteristics, and attitudes toward ecosystem services and biodiversity. For more details, see Supplementary Section H–J. As a result of the hierarchical clustering and the NbClust function, farmers were divided into two clusters (Table 1).

**Table 1:** Farmer clusters as a result of a factor analysis and hierarchical clustering

Variable	Farmer cluster 1 (f1) (480 participants)		Farmer cluster 2 (f2) (345 participants)	
Operation mode		+16% (full-time farming)		+21% (part-time farming)
Farming system		+15% (conventional)		+17% (organic)
Qualification		+16% (intermediate)		+9% (advanced)
Cropland area (size in hectare)		+17 hectares		–23 hectares
Nature activity: collecting non-timber forest products		Less: +15% (no)		More: +22% (yes)
Nature activity: watching wildlife		Less: +12% (no)		More: +18% (yes)
Sum of pursued nature activities		0		More: +1 activity
Considered importance: relation (farmer to nature)^a^		Higher: +7% (important)		Lower: –10% (important)
Considered importance: laws^a^		Lower: +9% (unimportant) & +6% (very unimportant)		Higher: +12% (important) & +7% (very important)
Considered importance: land used for income generation (points 0–100)		Higher: +4 points		Lower: –5 points
Considered importance: land used to support biodiversity (points 0–100)		Lower: –9 points		Higher: +12 points
Considered importance: land used to provide ecosystem services (points 0–100)		Lower: –6 points		Higher: +8 points

All reported cluster results are significant, with a *p*-value of 0.05. Grey backgrounds of arrows indicate the ten most significant variables per cluster. Higher/lower percentage values and points are to be understood as relative to the average of the entire sample

^a^For this survey question about the requirements for the success of nature protection in Bavarian agricultural landscapes, participants rated four items: subsidies based on success, subsidies for prescribed measures, stricter legal regulations, and personal relation of farmers to nature

### Qualitative Content Analysis

**Fig. 1:**
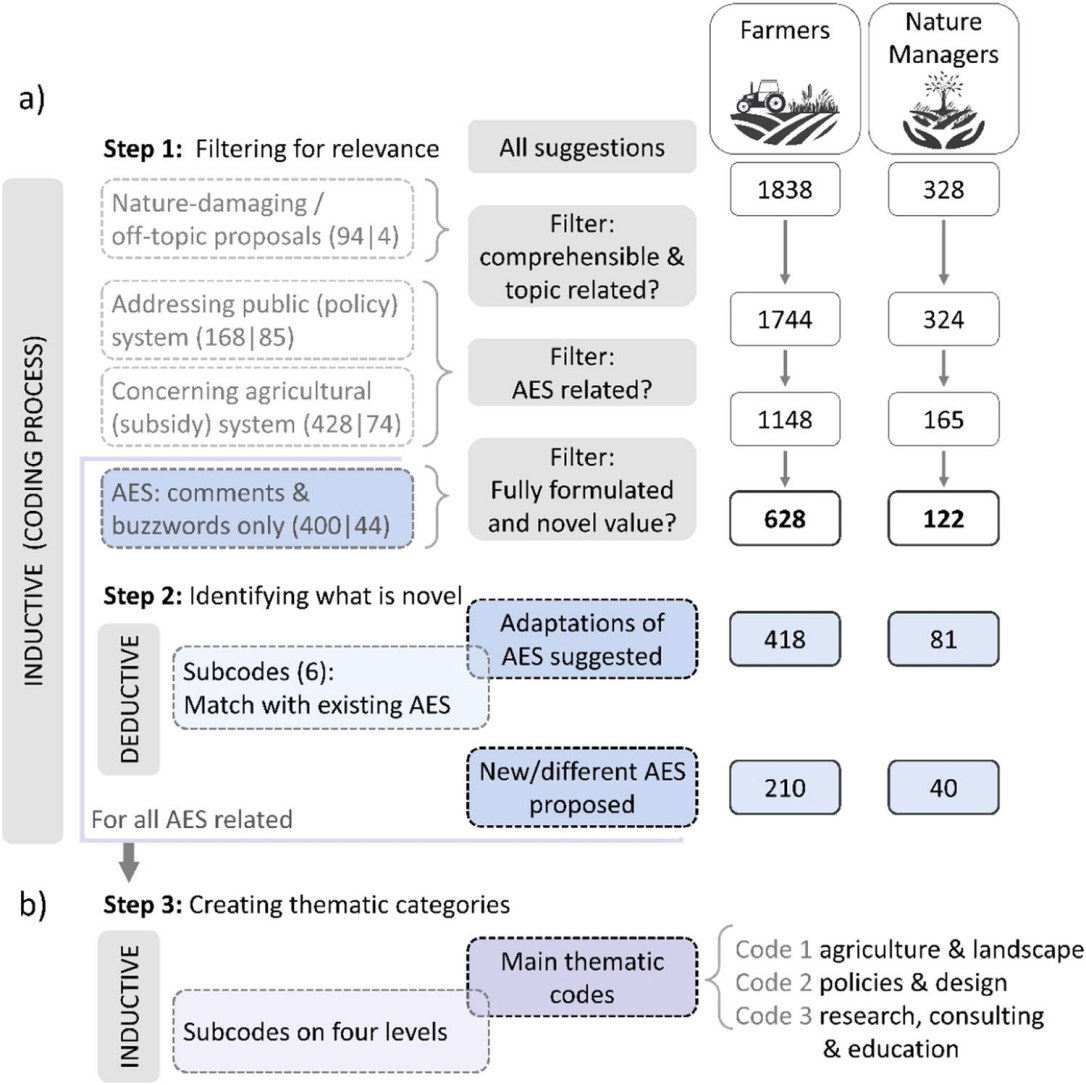
Main steps of the coding process of the suggestions for improved funding opportunities for nature conservation in agricultural landscapes. These included (**a**) filtering for relevance regarding agri-environmental schemes (AES) and (**b**) creating thematic categories. Numbers indicate the number of suggestions per step in the analysis for farmers (below the farmers’ icon) and for nature managers (below the icon). The coding process was generally inductive except for matching suggestions with existing AES

We performed a structuring qualitative content analysis (Mayring 2000; Kuckartz 2018) for each respondent group (farmers and nature managers). As our survey question did not specifically address AES but “funding” in general, we first had to filter relevant answers (65% of all coded segments, see Fig. 1). In our analysis, we focused on suggestions with a novel component, either (i) a new measure or concept, including suggestions for the general orientation of AES, or (ii) an adjustment of the existing AES and administration. Examples of the first are “*support for perennials*” or “*support based on success*” as a general way of compensation for AES. The latter contained ideas such as “*make crop rotation more flexible*” or “*extension of area setting of VNP*”. Generally, we chose an inductive coding process (Mayring 2000). We only used a deductive approach for matching the suggestions for adaptation of measures with AES already offered in Bavaria (Supplementary Tables A1 and A2). If participants’ answers addressed different topics in one suggestion, we split the suggestion into segments and coded each topic as a separate suggestion.

For the thematic categorization, we followed the concept of “theme codes” based on the main content or thought (Kuckartz 2018). Here, in addition to the two AES-related categories mentioned above, very short answers without any novel component (coded as “buzzwords and comments”) were also included. For the thematic coding, multiple codes could be assigned to one segment. For example, we coded the suggestion “*higher support for the cultivation of catch crops, instead of glyphosate application*” under two level 1 codes: *“*agriculture & landscape” and “policies & design*”* (see Supplementary Tables E1 and E2 for a detailed categorization of two examples). More information can be found in the Supplementary Section C–E, including a detailed description of each code (Supplementary Tables C1 and D1) and a frequency table (Supplementary Table D2). The described steps were conducted in the software MAXQDA Analytics Pro 2020 by VERBI.

## Results

**Fig. 2:**
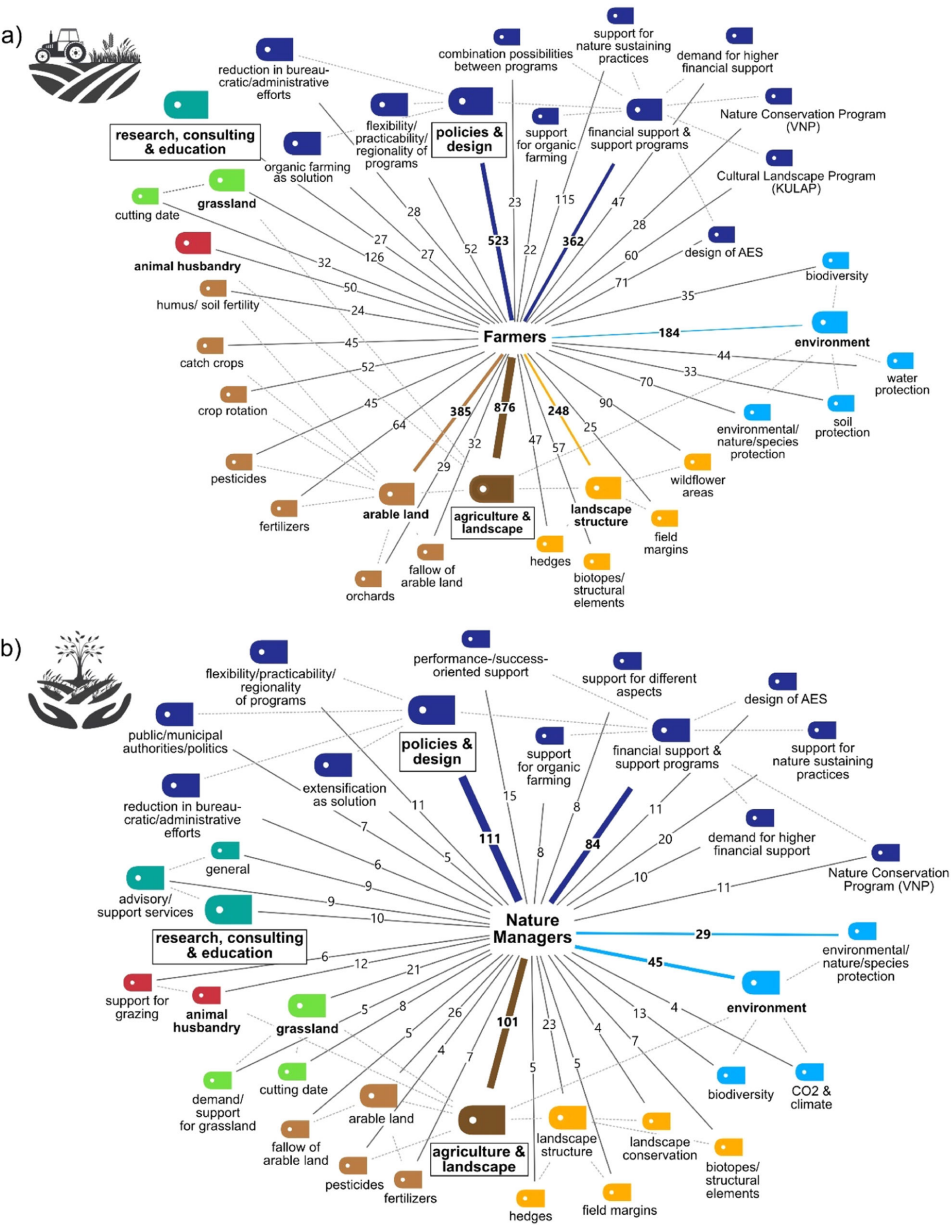
Code co-occurrence model showing the most frequent codes for (**a**) all farmers (accounting for at least 2% (*n* > 22) of all relevant answers (*n* = 1148) (see Supplementary Table D2, for code frequencies differentiated for the two farmer clusters) and (**b**) nature managers (accounting for at least 2% (*n* > 3) of all relevant answers (*n* = 165)). Sizes of tags display the level within the code system (level 1 = biggest tag, level 3 = smallest tag). Level 4 is not displayed (see Supplementary Table D1 for details on coding levels). Colored lines indicate the five codes with the highest frequencies. Numbers indicate the count of coded segments. Dashed lines indicate which codes (smaller size) are subordinate to the respective code (larger size)

**Fig. 3:**
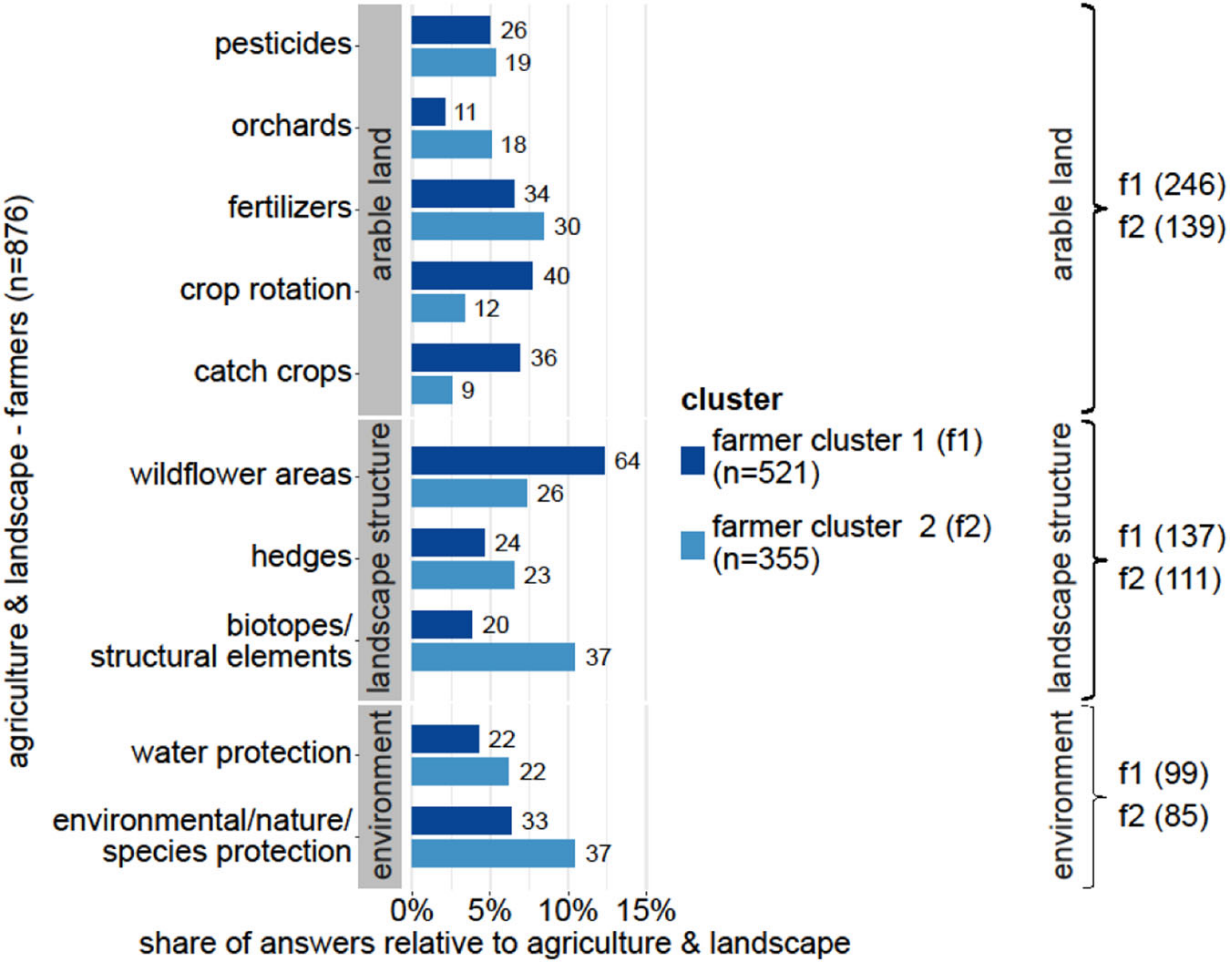
Differences between the farmer clusters (f1 and f2) for the code “agriculture & landscape” (level 1). Displayed are the relative shares of the three most frequent sub-grouped thematic codes (level 2): arable land, landscape structure, and environment. Numbers behind bars indicate absolute numbers (counts) of coded segments. Codes are only displayed if they take a share of at least 5% within “agriculture & landscape” (for at least one of the clusters). The numbers on the right indicate the counts per cluster for code level 2. See Supplementary Fig. F1, for a comparison of the other frequent level 1 code, “policies & design”

The thematic focus of AES-related suggestions by farmers and nature managers showed many overlaps (Fig. 2). However, farmers put more emphasis on concrete measures concerning agriculture and landscape. In contrast, nature managers focused more on policies and general design (Fig. 2). Farmer cluster 1 (f1) had a higher share of suggestions for arable land, while farmer cluster 2 (f2) highlighted landscape structures (Fig. 3). New and substantially different approaches for AES comprised 18% of AES-related suggestions for farmers and 24% for nature managers. Suggested adaptations for existing AES comprised 36% of the AES-related suggestions for farmers and 49% for nature managers.

### Farmers’ Suggestions for AES

#### New and substantially different approaches for AES

Several respondents mentioned measures related to humus formation. Proposals included the use of microorganisms, humus-benefitting crop rotations, the systematic incorporation of biomass material (f1), as well as perennials and catch crops for humus formation (f2). Another dominant topic was the reduction of fertilizers. Ideas were related to supporting efficient yet minimal fertilizer application, e.g., 30% lower than official recommendations (f1). Participants also addressed nitrogen-stabilizing fertilizers, nitrogen-efficient farms, and the Controlled Uptake Long Term Ammonium Nutrition (CULTAN) fertilization method (Deppe et al. 2016) (f1). Especially farmers in f2 demanded less (support for techniques for) manure application. Farmers in both clusters gave suggestions addressing (subsidies for) lower/minimal herbicide usage, e.g., via fungicide-resistant varieties, beneficial animals (f1), or mechanical weed control.

Furthermore, various farmers (particularly in f2) suggested support according to farm or field-related criteria such as parcel size (the smaller, the more money) or livestock units, e.g., support for farms with less than 50 livestock units (f1) or support according to the soil fertility (f2) or level of environmental protection (f1). Result-based compensation was another frequently mentioned topic (more in f2). Moreover, respondents in f1 requested support for extensive crops, e.g., a new measure with only 33% intensive crops like wheat, corn, and sugar beet. Additionally, ideas for integrated farming (measures) consisted of reduced tillage for erosion protection in combination with reasonable plant protection and measures that can be easily implemented and integrated into the regular agricultural business. Some respondents mentioned the integration of meadows between crop fields to achieve a more diverse landscape and expressed a desire to strengthen hybrid agriculture by combining the benefits of conventional and organic agriculture. Alternating mowing practices were also suggested. Several farmers in f1 suggested (support for) reduced soil cultivation, including no-till. In contrast, the topics (money for) agroforestry and three-field crop rotation had slightly more answers in f2. Less frequently, ideas addressed cultivating mixed crops in stripes on one field and using smaller/lighter machines. A detailed description of selected suggestions is presented in Supplementary Section F.

#### Adaptations for existing AES

A strong focus was set on increased financial support for the programs, single measures, and organic farming (Supplementary Fig. F1 and Supplementary Table F1). Farmers in cluster f1 expressed a strong wish for (more) flexible and practical administration, especially a lower bureaucratic load (Supplementary Fig. F1). Farmers demanded more options to adapt to particular circumstances, removal of fixed dates, and the possibility to switch measures every year. One farmer proposed that “*local authorities should obtain decision-making scope. [KULAP]-measures that fit in the wet Allgäu [region in Bavaria] do not necessarily fit in the dry locations in Franconia [region in Bavaria]. […]*” or that “*the financial incentive for implementing the measures must be adapted according to a conversion key per municipality, e.g., 5% of the municipal area should be wildflower areas. In case this is not reached, the financial incentive must be increased. […]*” (f1). Furthermore, respondents supported adaptability according to local (weather) conditions. One farmer wrote, “*Make KULAP more flexible. Not every year is the same in terms of weather. That means if my oilseed rape is not sprouting in autumn because it didn’t rain, but I am dependent on the oilseed rape due to KULAP-reasons, I would like to cultivate less this year and then more in the next year. There should be a balancing (Saldierung) within the five-year commitment period*.”. Additionally, respondents in both clusters desired less complex programs.

Farmers in both clusters requested additional consultation opportunities. Suggestions included improved advice for farms switching to organic production (f2) and individual consulting for climate and nature protection (f1). More farmers in f2 demanded enhanced information sharing (roundtables, workshops) about AES-related topics, connecting conventional and organic farmers. Participants stressed the need for more individual, farm-specific measures and programs, e.g., to support (ecological) farm concepts (f1). Especially farmers in f2 demanded better long-term planning and legal certainty. In addition, farmers across clusters expressed support for combining measures, especially in the context of organic farming.

Specific recommendations for several existing measures were given (see Supplementary Table F1). For KULAP and VNP, adaptations for the cutting date were emphasized, particularly in f1. Often, a lower cutting frequency was endorsed, mostly in f2. Moreover, some farmers requested measures for more insect- and animal-friendly mowing techniques, e.g., with cutter bars, without area restriction (i.e., outside protected areas) (f2), and no/less mulching. Suggestions for the LNPR (mostly by f2) included specific measures targeting wildlife and birds, improved support for bog meadows (f1), more regional species protection programs (f2), and improvements for insects.

#### Comparison of thematic focus per farm cluster

Crop rotation, catch crops, and wildflower areas had a higher representation in f1 (Fig. 3). In contrast, f2 showed higher shares for environmental protection, structural elements, fertilizer use, and hedges (Fig. 3). Relative to cluster f1, a larger proportion of the respondents in f2 mentioned funding and support programs (Supplementary Fig. F1a). Within this category, farmers in cluster f2 gave more answers related to the support of organic farming, the VNP, or the design of AES. On the other hand, support for nature-sustaining practices and the KULAP were more prominent in f1 (Supplementary Fig. F1b). The flexibility of programs was more important for farmers in cluster f1, while reducing bureaucratic efforts and organic farming as a solution played a more prevalent role in f2 (Supplementary Fig. F1a).

### Nature Managers’ Suggestions for AES

#### New and substantially different approaches for AES

One important aspect mentioned by nature managers was result-based subsidies. Examples included subsidies according to the existing biodiversity level, sustainability or ecological criteria, or the extensification level. Some nature managers proposed a bonus for the successful reproduction of meadow-breeding birds or support for biodiverse habitats, e.g., a payment of 200 €/ha if partridges are detected or compensation for discovering a bird clutch of target species. Other responses addressed spaces for biodiversity and specific support programs focusing on umbrella species.

Some suggestions expressed support for the structural diversity of farm systems. In contrast to the farmers, less focus was put on the distinction between organic and conventional. Respondents mentioned support for sustainable management systems, historical management methods, regenerative agriculture, and areas targeting a specific purpose, e.g., climate farming. Other ideas involved subsidies to maintain soil fertility and a bonus for general extensification (focusing not only on areas with high environmental value). Nature managers mentioned three-field crop rotation and treating manure to reduce ammonia emissions. Suggestions targeting future trends were the support for new technologies like drones and climate-adapted land use concepts, including the support of climate-resilient crops.

#### Adaptations for existing AES

Like farmers, nature managers focused on adaptations related to the programs’ orientation and administration (Supplementary Table G1), with a strong focus on monetary support. Multiple respondents demanded much stronger financial support (not only compensation), particularly for organic farming, the VNP, and grazing. Other ideas included subsidies for small areas (missing in KULAP), support independent of the area setting, and farm and field-specific measures to support species and biotopes. In this context, a better adaptation of the VNP to single species or landscapes was demanded. Furthermore, nature managers endorsed longer-term measures and environmental assessments.

Nature managers frequently expressed support for flexibility, practicality, and a stronger focus on regional characteristics. Suggestions included adaptations according to the climate or species distribution and more (financial) decision-making scope regarding single measures. One stated: “*[…] on the discovery of meadow-breeding birds clutches on an intensive meadow, farmers need replacement. If no meadow-breeding birds are breeding on a [VNP] meadow, it would be good […] to exceptionally mow these before the fixed cutting date*.”. However, some respondents discussed stricter rules with more/improved inspections and strict payment reductions in cases of non-compliance. Like farmers, a reduced bureaucracy was requested, including a less complicated allocation of subsidies and yearly payments. Other wishes were faster implementable measures and an improved proportionality between administrative/control efforts and the resulting effectiveness.

Some nature managers wished for more/improved information and consulting, e.g., through biodiversity consultants and qualified personnel (regarding agriculture, not only nature protection) or nature conservation authorities. Respondents also suggested more consulting and information about environmentally-friendly cultivation. In this context, demand for more personnel was expressed.

Like farmers, ideas for specific AES (see Supplementary Table G1) included enhanced opportunities to combine the KULAP, the VNP, and organic farming. Nature managers noted the need for flexible cutting dates with yearly adaptations based on the vegetative state and the reduction of the cutting frequency, e.g., via supporting a maximum of 2 cuts. Furthermore, better options for embedding ecological measures within “normal” agricultural landscapes as well as more funding for area-linked animal husbandry, especially grazing systems, were mentioned. Other adaptations addressed the LNPR, which included support for targeted land acquisition for Bavaria’s biotope network (insufficient current support, e.g., by the LNPR and VNP). It was also suggested to “*support […] landscape conservation areas with 90 to 100% [of costs] - otherwise, it will always be in the interest of the Associations for Landscape Management (Landschaftspflegeverband) to have as few conservation areas as possible and not to hire new personnel […]*”. Besides, more consequent protection and revitalization of bogs were demanded.

### Comparison between Suggested Measures and AES Participation Data

In Bavaria, three KULAP measures dominated the area cover in 2020 (Supplementary Table H1): Low emission distribution of liquid manure/organic fertilizers, organic farming, and crop rotations with legumes. In the survey answers, the latter two were well represented. Similarly, the abandonment of any fertilizers and pesticides, which shows the largest area within VNP (Supplementary Table H1), was a recurring topic in this study. Furthermore, extensified mowing of valuable habitats and cutting date measures saw high participation in Bavaria and were frequently mentioned by respondents of our study. Suggestions for restricting the number of livestock per grassland area were less dominant in our sample, despite large areas enrolled in the extensive grassland use with 1.4 or 1.76 livestock units per hectare. Also, mulch seeding, catch crops, and orchards had high area cover in 2020, though respondents did not often address mulch seeding.

Few answers reflected on summer grazing (KULAP), a measure with many applications (Supplementary Table H1). The high application numbers for extensive grassland use along water bodies aligned with the number of participant suggestions. Another contrast between the participation statistics and the results was apparent for wildflower areas (not among the top five measures implemented in Bavaria until 2020, although related suggestions were manifold). A further discrepancy relates to the low application numbers for subsidies related to the area provision for establishing structural elements despite the high number of responses in our study. In 2020, only five people applied for this measure, and only six applications in Bavaria were submitted for the investment measure (establishment of structural elements) since 2016. However, the application numbers for the renewal of hedges and field shrubs were high (StMELF, E-mail communication, March 9th, 2022). Additionally, old grass strips (supported since 2020) and support for small fields increased over the last years, and farmers and nature managers addressed the importance of both.

## Discussion

### Differing Focus between Farmers and Nature Managers

Clear differences exist between the suggestions made by farmers and nature managers. Nature managers focused on agricultural policies, support programs, and municipal authorities, whereas farmers’ suggestions centered around topics linked to agricultural practices and landscape structure. This might be explained by the fact that nature managers are more often included in policy-making and responsible for their (local) implementation (Fleury et al. 2015; Prager and Freese 2009; StMUV 2021). Furthermore, farmers emphasized farming-related practices for arable land, while nature managers strongly focused on environmental topics, particularly on nature and species protection as well as biodiversity.

However, in contrast to the study results by Sattler and Nagel (2010), farmers in the present study emphasized nature and species protection rather than water and soil protection. The higher awareness about factors that are indirectly linked to production (i.e., species protection) compared to direct factors (soil, water) is likely a result of the high participation rate in AES (and related positive environmental attitudes) within the farmer sample (Lastra-Bravo et al. 2015). The strong focus of farmers on arable land is underpinned by Włodarczyk-Marciniak et al. (2020), who found that farmers consider productive agricultural landscapes such as cultivated fields particularly important. Similarly, farm advisors surveyed in the study by Riley (2016) explained that decisions to adopt measures are highly driven by economic concerns and the possibility of easily integrating measures into the ongoing farm business. That farmers’ incomes depend (partly) on the productivity of their land explains their focus on productivity in their suggestions (Wossink and van Wenum 2003). This may explain why ideas for success-oriented remuneration, where income is not guaranteed, were more prevalent among nature managers than farmers. However, both groups frequently addressed subsidies, which indicates the importance of compensation for productivity losses when implementing AES (Defrancesco et al. 2008; Lastra-Bravo et al. 2015).

Mentions of nature and species protection within the answers of the nature managers are connected to one of their primary work goals (StMUV 2021). This trend toward discussing more environmental topics can be corroborated by the results of Maas et al. (2021), who found that scientists attribute higher importance to biodiversity and habitat protection than farmers. Although nature managers may not be scientists, the majority in our sample hold a university degree, indicating a scientific background. Similarly, in a study on ecosystem services perceptions in Bavaria, Germany, Thiemann et al. (2022) found that habitat services were perceived to be very important for over 90% of the nature managers that responded to the survey.

Contrary to Maas et al. (2021), this study did not find that nature managers consider landscape elements like wildflower areas or buffer strips more important relative to farmers. We found that more than 10% of all answers related to agriculture and landscape made by farmers mentioned wildflower areas. However, the dominance is neither reflected in the area enrolled in this measure nor in the number of applications. An explanation could be linked to the political context. Following a 2019 referendum in Bavaria that focused on (insect) biodiversity (Hartmann et al. 2021), a new and highly renumerated measure of annual wildflower areas was offered in 2020. This measure was well received but limited to 6 ha per farm, explaining the many related comments. Nature managers’ low support for wildflower areas could be connected to the controversy about their effectiveness (Dietzel et al. 2019). Nature managers also rated consulting and education as slightly more important than farmers. This is likely because some nature managers advise farmers and are confronted with existing knowledge gaps and areas where advice is needed (Hejnowicz et al. 2016).

### Differences within Groups of Farmers

Our identified differences between the two farmer clusters are supported by the findings of Maas et al. (2021). The authors of that study also found that farmers with a higher education level as well as organic and female farmers, assigned higher values to the importance of biodiversity and ecosystem services. Our farmer cluster f2, with more organic farmers, showed a tendency towards environmental topics and landscape structures. Cluster f1 had a slightly stronger focus on farming practices in arable land (Fig. 3). One exception were wildflower areas, which were more prominent in f1. A reason for this might be that the higher education status of cluster f2 promoted a more reflected view on the debated effects of wildflower areas for biodiversity enhancement (Dietzel et al. 2019). Additionally, farmers in cluster f1, with a higher proportion of full-time farmers, might have perceived the high compensation rate for wildflower areas (600 €/ha) as a decisive factor for the scheme’s attractiveness. Furthermore, the higher share of full-time farmers may explain the tendency toward measures on productive areas, e.g., catch crops and crop rotation, which are the most discussed practices in f1.

The lower presence of answers for crop rotation and catch crops in f2 might result from these measures being part of regular organic farming practices (Council of the European Union 2007; Barbieri et al. 2017). Additionally, despite a high (planning) effort for the initial set-up, these measures are classified as having lower requirements regarding management restrictions and only medium environmental benefits (Sattler and Nagel 2010; art 2016). The positive attitude toward biodiversity in cluster f2 likely triggered the proposal of more effective measures, such as establishing different structural elements (Batáry et al. 2015; Dietzel et al. 2019). Mack et al. (2020) corroborate these results by pointing out that organic farmers might have a lower barrier to implementing conservation measures and, therefore, are less afraid of lost revenue. However, one aspect often addressed in f2 concerning measures on productive land was the desire to re-establish the possibility of combining organic farming with diverse crop rotation. The additional remuneration of combining these measures seems to play a critical role in fostering organic farming and should be reconsidered, given the goal to achieve 30% organically cultivated land in Germany in 2030 (BMEL 2022; Koch 2020).

We observed discrepancies between the uptake of measures in Bavaria and the focus of study respondents. Many suggestions addressed biotopes and structural elements, but participation rates were low (Section “Comparison between suggested measures and AES participation data”; StMELF, E-mail communication, March 9^th^, 2022). However, increasing the uptake of measures targeting landscape elements would benefit the creation of habitats (Morelli 2013; Graham et al. 2018). Especially farmers in f1 discussed the need for more flexibility and practicality regarding the implementation of structural elements and better compensation. The fact that measures for hedges have been accepted well underpins the general willingness of farmers to establish structural elements if underlying requirements are suitable and measures are adapted.

### Selection of the Most Innovative Measures and Recommended Adaptations of AES

Despite these differences, many overlaps were found between and within the two respondent groups (farmers and nature managers). This section presents some recommendations for policy adaptations based on innovative suggestions and dominant topics raised by both groups. However, some suggestions might be conflicting and need careful design to find the best possible compromise.

#### Result-based AES

Participants highlighted result-based measures, which have been widely researched (Burton and Schwarz 2013; Schroeder et al. 2013). One well-accepted measure from the KULAP links payments to the occurrence of a minimum of four indicator species (LfL 2018). Similar measures exist in other parts of Germany, e.g., within the FAKT program in Baden-Württemberg (MLR 2022). Our study suggests that financial security is vital for financially dependent farmers. Similarly, Sattler and Nagel (2010) identified risks as a major influence on adopting schemes. The imposed uncertainty of result-based payments – due to their dependency on the delivery of goals, which are also influenced by natural conditions – needs to be addressed in future schemes (Westerink et al. 2008; Burton and Schwarz 2013).

#### Reduction of fertilizer and pesticide use

Respondents expressed demand for measures supporting the reduction of fertilizer and pesticide use. The importance is reflected in the currently high uptake of related AES (within VNP) in Bavaria. One other solution from Baden-Württemberg could be precision farming. This uses techniques like remote sensing and geoinformation systems to adapt inputs (fertilizer, pesticides, water) exactly to crop needs. This results in environmental and economic benefits (Balafoutis et al. 2017), but specific devices and knowledge are needed (Finger et al. 2019). Another less cost-intensive idea suggested by farmers could be the compensation for fertilizer applications below recommended values, though the problem of controlling compliance would need to be solved. Introducing a measure abandoning herbicides in crop cultivation in the KULAP in 2021 was a promising step. A useful addition could be to support the abandonment of pesticides or fertilizers on the entire farm (not yet meeting the requirements of organic farming) or single fields, as offered in Baden-Württemberg. With the post-2020 CAP reform, new voluntary eco-schemes are offered for one-year periods. Since 2023, this includes abandoning chemical and synthetic pesticides in Germany (Latacz-Lohmann et al. 2022; Runge et al. 2022). The use of biological pest control in the form of beneficials was also suggested. For example, the use of trichogramma in corn, introduced in 2021 in Bavaria, was well accepted (rank 19 of 39 measures; European Commission 2014), indicating the potential for similar measures. Additionally, compensation for mechanical weed control could be a viable solution (Kunz et al. 2015; Sims et al. 2018).

#### Humus formation

Survey participants frequently demanded measures on humus formation. Specific ideas included support for perennial crops, incorporating biomass material, separate measures compensating no-till or reduced till practices, more humus-benefitting catch crops, and suited crop rotations with legumes. The effectiveness of these suggestions is well supported (Küstermann et al. 2013; Beniston et al. 2014; Cates et al. 2016) and recognized on a political level (NABU 2013; LfL 2018; Flessa et al. 2019). One humus-benefitting measure, which farmers criticized due to the strict area limitations, is the conversion of cropland into grassland. Permanent grassland and extensive grassland management can build humus (Wiesmeier et al. 2013). Yet, substantial areas of permanent grassland have been lost in Bavaria (Haensel et al. 2023). The removal of area limitations could counteract the decreasing trend of area enrolled in the respective KULAP measure.

#### Grassland measures

Despite high enrollment in grassland AES in Bavaria, study participants focused little on grassland topics, suggesting fewer adaptation needs by respondents. Grazing and cutting dates received the most remarks. For cutting date measures, increased flexibility regarding local conditions was one of the main requests. Ruto and Garrod (2009) showed that flexibility regarding measures increased AES participation. As high demand was expressed for cutting date measures and animal-friendly mowing techniques, existing measures in the VNP should be extended, including an increased budget. Nevertheless, cutting date and frequency measures require compromises between flexibility and environmental targets, as, e.g., the deferral of mowing dates by only one day influences the ecological effectiveness (Latacz-Lohmann and Breustedt 2019).

#### General set-up of AES

Many respondents supported adopting measures according to stakeholders’ needs. This could include increased remuneration, reduced bureaucracy, more flexibility, variable contract length, or more effective (species) targeting of programs. Previous research has also highlighted these topics (Defrancesco et al. 2008, 2018; Wrbka et al. 2008; Ruto and Garrod 2009; Meyer et al. 2015). Lean bureaucracy and a better understanding of implementation practicalities can decrease administrative transaction costs (Falconer and Whitby 2000). This could cover increased costs needed for personnel providing better support and regionally specific programs. It is important to note that some of the discussed aspects for improved AES might be conflicting. This is particularly true for the demand for a reduced bureaucratic burden and the wish for targeted measures adapted to local conditions. For example, more flexible cutting dates would require some margin of discretion to assure equality among the farmers in compliance with the measures, which would, in turn, likely decrease practicality on the administrative side. Another example is the suggestion to reduce fertilizer use to 30% below the recommended value. Here, administrative problems would need to be solved, such as the definition of locally differing thresholds or the problem of controlling compliance. This calls for well-designed measures, where increased flexibility or better targeting does not result in a much higher bureaucratic burden for farmers and administrative offices.

Assuming a dichotomy between organic and conventional farming fails to recognize different levels of ecological practices within conventional agriculture (Höglind et al. 2021). This was reflected in the participants’ demand for more nuanced support regarding various farm structures and sizes, such as regenerative agriculture, input-efficient farms, or farms that partly fulfill organic criteria. Furthermore, the wish to increase support for small field structures could be fulfilled by lifting requirements regarding the area setting for this measure in the VNP. To ensure the success of different farm structures, the desired establishment of individual financial and advisory support could have a positive influence (Riley 2016; Birge and Herzon 2019). Beyond this, suggestions illustrate the wish to support a variety of newer, innovative, and also traditional practices, including permaculture, agroforestry, agro-photovoltaics, drones, three-field crop rotation with fallow, and old cultivars. The new CAP eco-schemes incorporate measures targeting agroforestry and fallow (StMELF 2023), but there is still room for further integration of practitioners’ suggestions.

### Methodological Considerations and Limitations

A major limitation lies in potential biases in our sample of respondents. Our sample over-represented farmers participating in AES and organic farming. The similar environmental attitudes arising from this might explain why differences within the group were not so high (Defrancesco et al. 2008). This could also have impacted the cluster analysis. Here, the explained variance of the first two dimensions and the total explained variance of around 58% were in line with similar studies (Beltrán-Alcrudo et al. 2018; Hair et al. 2019). Another factor influencing the results could be selection bias, especially for those surveys distributed online due to the COVID-19 pandemic (Eysenbach and Wyatt 2002; Meho 2006). In contrast to the large farmer sample, the sample size of nature managers was relatively small. Yet, a wide range of institutions were represented, and the risk of a self-selection bias is expected to be less pronounced, as interest in nature protection is potentially a prerequisite to working or engaging in this field.

Another limiting factor could be the wording of the main survey question. The open-ended question did not explicitly address AES (see Section “Survey of farmers and nature managers”). This explains the broad range of (agricultural) issues discussed by respondents. For example, the assumed links to AES of the answer “bureaucracy reduction” could have been intended by respondents in a broader sense. However, almost 90% of farmers were presented with a choice experiment showing examples of AES before the open-ended question. This makes it plausible that the assumed link between given answers and AES held true in most cases. Due to our survey format, stakeholders likely provided input based on their existing values, beliefs, and experiences. In order to capture suggestions that are still vague or to promote the creative development of new ideas, other methods, such as qualitative interviews or transdisciplinary group processes, would be necessary.

The study illustrated suggestions from stakeholders to foster the use of AES in a central European region. However, some aspects of this study are very context-specific and only have regional relevance for Bavaria. Yet, the study results point to the vital need to include stakeholders’ perceptions of AES in the design of measures and to create more flexible and practical measures. These results are in line with findings by other studies in different regions. For instance, Schulze and Matzdorf (2023) analyzed stakeholders’ attitudes toward AES in eastern Germany, characterized by much larger farm areas and a different historical development. They also concluded that various stakeholders should be involved in designing conservation measures and creating (sub) programs that fit the needs of specific land managers.

## Conclusion

The goal of our study was to assess farmers’ and nature managers’ recommendations and adaptations for agri-environmental schemes. Our results show that both groups have various ideas for improving current schemes and are willing to share their experiences. Policymakers should aim for a more active integration of stakeholders across the policy-making process. In addition, this study provides evidence that farmers’ needs depend on underlying background conditions, including social, demographic, and economic factors, as well as farm characteristics. This suggests that a more nuanced design of AES, with a more regional adaptation as well as different levels of flexibility, practicability, and nature protection value, is important in developing future AES. With the new CAP eco-schemes having started in 2023, some of the discussed measures, such as a shorter commitment period, have been put into action. However, many practitioners’ suggestions are not reflected in the schemes yet. Thus, there is still ample room for improvement, both in terms of practicality and impact. Additionally, future research should focus more on considerations of nature managers, as their views are particularly helpful in connecting species protection and farming better.

Specifically for the farmers, the study reveals deficits in the knowledge about ecosystem services. Farmer suggestions often targeted familiar farming practices based on external inputs rather than using benefits provided by ecosystem services (Bommarco et al. 2013). This implies that better ways of incorporating benefits from nature protection and ecosystem services into the subsidy system could be found. Additionally, raising farmers’ awareness about ecosystem services and how their enhancement can promote agricultural performance would be a promising step.

### Supplementary information

Supplementary Information
