# Supplementary material for: Improving Agri-environmental Schemes: Suggestions from Farmers and Nature Managers in a Central European Region
Source: Environ Manage. 2023 Dec 18;73(4):826–40. doi: 10.1007/s00267-023-01922-w (PMC10972928; doi:10.1007/s00267-023-01922-w)
Supplement: Supplementary file 1 — Supplementary Information [file 267_2023_1922_MOESM1_ESM.pdf]

Supplementary Information

For manuscript "Improving agri-environmental schemes: Suggestions from farmers and nature managers in a central European region" (Environmental Management)

Authors: Manuela Zindler[1], Maria Haensel[1†], Ute Fricke[2], Thomas M. Schmitt[1], Cynthia Tobisch[3], Thomas Koellner[1]

[1] University of Bayreuth, Professorship of Ecological Services, Bayreuth Center of Ecology and Environmental Research (BayCEER), Universitätsstraße 30, 95447 Bayreuth, Germany
[2] Department of Animal Ecology and Tropical Biology, Julius-Maximilians-University, Am Hubland, 97074 Würzburg, Germany
[3] Institute of Ecology and Landscape, Weihenstephan-Triesdorf University of Applied Sciences, Am Hofgarten 4, 85354 Freising, Germany.

† Corresponding author:
Maria Haensel, University of Bayreuth, Professorship of Ecological Services, Bayreuth Center of Ecology and Environmental Research (BayCEER), Universitätsstraße 30, 95447 Bayreuth, Germany, maria.haensel@uni-bayreuth.de;

Manuela Zindler: https://orcid.org/0009-0002-1557-9193
Maria Haensel: https://orcid.org/0000-0003-4530-8968
Ute Fricke: https://orcid.org/0000-0002-5284-4518
Thomas M. Schmitt: https://orcid.org/0000-0002-7287-6648
Cynthia Tobisch: https://orcid.org/0000-0002-2039-1462
Thomas Koellner: https://orcid.org/0000-0001-5022-027X

List of Tables (Supplementary Information)

Table A1. Measures of the Bavarian KULAP since 2015. 3
Table A2. Measures of the VNP since 2015 5
Table B1. Institutions contacted during the E-mail survey for the nature managers and additional remarks 7
Table B2. Counts of respondents working/engaging in the respective organization. 8
Table C1. Detailed description of the categories, including subcategories, for the differentiation of new and existing ideas, and the number of suggestions in each category for nature managers (nat), farmer cluster 1 (f1), and farmer cluster 2 (f2). 9
Table D1. Detailed description of all thematic categories. 13
Table D2. Thematic categorization system with frequencies of coded segments. 27
Table E1. Exemplary coding for the suggestion of example 1. 33
Table E2. Exemplary coding for the suggestion of example 2. 33
Table F1. Detailed description of selected farmer suggestions 35
Table G1. Suggested adaptations by nature managers for the KULAP and the VNP. 37
Table H1. The top five measures regarding the area enrolled and number of applications for the KULAP and the VNP for the year 2020.. 38
Table I1. Variables with respective questions from the LandKlif survey used for the cluster analysis of the farmers. 39
Table K1. The ten most influencing qualitative variables for each farmer cluster. 48
Table K2. Socio-demographic and farm characteristics per each farmer cluster. 49

List of Figures (Supplementary Information)

Fig. F1. Relative share of the most dominant codes within (a) policies & design and (b) financial support & support programs as a subcode of policies & design for the group of farmers per cluster.35
Fig. J1. Farmer clusters as a result of the hierarchical clustering and the NbClust function. 44
Fig. J2. Contribution of each variable to dimension one.. 44
Fig. J3. Contribution of each variable to dimension two (farmers). 45
Fig. J4. Contribution of the quantitative variables to the first two dimensions. 45
Fig. J5. Contribution of the qualitative variables to the first two dimensions. 46
Fig. J6. (a) Dendrogram computed by the HCPC function. (b) Bar plot with inertia gain computed by the HCPC function, showing the division into the two farmer clusters. 46
Fig. J7. Optimal number of clusters according to the NbClust function.. 47

A. Measures of the Bavarian KULAP and the VNP since 2015

Table A1. Measures of the Bavarian KULAP since 2015. Rows that are colored in green mark measures that are applied on grassland. Rows that are colored in brown mark measures that are applied on cropland. Rows that are white refer to management practices that can be applied to different types of land. Source: StMELF, 2020b, 2020c.

	Code	Measure description	Target area	Intro-duced[1]	Unavail-able[2]
Organic farming	B10	Organic farming	Entire farm	2015	
Climate protection	B19	Extensive grassland use for gazing stock eating roughage; maximum 1.0 LSU	Branch related	2020	
	B20	Extensive grassland use for gazing stock eating roughage; maximum 1.4 LSU	Branch related	2015	2016
	B21	Extensive grassland use for gazing stock eating roughage; maximum 1.76 LSU	Branch related	2015	2016, 2021
	B22	Extensive grassland use for gazing stock eating roughage in alpine areas; maximum 1.4 LSU	Branch related	2015	2016
	B23	Extensive grassland use for gazing stock eating roughage in alpine areas; maximum 1.76 LSU	Branch related	2015	2016, 2021
	B25	Low emission distribution of liquid manure/organic fertilizers using own equipment	Farm business related	2015	
	B26	Low emission distribution of liquid manure/organic fertilizers across multiple farm businesses	Farm business related	2015	
	B28	Conversion of cropland into grassland along water bodies and in other sensitive areas	Single areas	2015	2016
	B29	Conversion of cropland into grassland along water bodies and in other sensitive areas – bog areas	Single areas	2015	
Soil & water protection	B30	Extensive grassland use along water bodies and in other sensitive areas	Single areas	2015	2016
	B32	Riparian strips and erosion protection strips in red areas according to the AVDüV (fertilizer ordinance); 7-30 m width	Single areas	2019	
	B33	Riparian strips and erosion protection strips in red areas according to the AVDüV (fertilizer ordinance); 13-30 m width	Single areas	2019	
	B34	Riparian strips and erosion protection strips; 6-30 m width	Single areas	2015	
	B35	Winter soil cover with catch crops (and undersown crops)	Single areas	2015	2016-2019, 2021
	B36	Winter soil cover with wild plant seeds (*Wildsaaten*)	Single areas	2015	2016
	B37	Mulch seeding for row crops	Single areas	2015	2016, 2021
	B38	Strip/direct seeding for row crops	Single areas	2015	2016
	B39	Abandonment of intesive crops in areas sensitive for water resourcse management	Single areas	2021	
	B62	Abandonment of herbicides in crop cultivation	Single areas	2021	
	B63	Use of trichogramma in corn	Branch related	2015	

Biodiversity	B40	Maintenance of biodiverse grasslands	Single areas	2015	2016, 2017
	B41	Extensive grassland use	Single areas	2015	2016, 2017
	B42	Establishment of old grass strips	Single areas	2020	
	B43	Diverse crop rotation with flowering crops	Branch related	2020	
	B44	Diverse crop rotation with protein crops (legumes)	Branch related	2015	2016, 2017
	B45	Diverse crop rotation with large grain legumes	Branch related	2015	2016, 2017
	B46	Diverse crop rotation with old crops	Branch related	2015	2016, 2017
	B47	Annualy changing wildflower areas	Single areas	2015	2016
	B48	Wildflower areas along forest margins and in the field	Single areas	2015	
	B61	Wildflower areas along forest margins and in the field (maximum 30% arable land)	Single areas	2020	
Cultural landscape	B50	Hay milk – extensive feed extraction	Branch related	2015	
	B51	Mowing of steep meadows	Single areas	2015	2016
	B52	Continuous management of recognized alps (alpine regions)	Single areas	2015	2016
	B55	Viticulture on steep slopes and terraces	Single areas	2015	2016
	B57	Orchards	Single areas	2015	2016
	B58	Extensive pond management	Single areas	2015	2016
	B59	Provision of area for the permanent establishment of structural and landscape elements	Single areas	2016	
Investment measures	B59	Establishment of structural and landscape elements	Single invest-ment	2016	
	B49	Renewal of hedges and field shrubs	Single invest-ment	2016	
	B56	Establishment of stone walls in viticulture	Single invest-ment	2016	

1. Only the measures after the restructuring of the KULAP in 2015 are shown. Measures with the year 2015 might have existed in a similar way before 2015.

2. Unavailable means that the respective measure was not open for new applications in the indicated year.

Table A2. Measures of the VNP since 2015. Rows that are colored in green mark measures that are applied on grassland. Rows that are colored in brown mark measures that are applied on cropland. Rows that are colored in blue mark measures for ponds. Rows that are white refer to management practices that can be applied to different types of land. Source: StMELF, 2020c, 2020d.

	Code	Measure description
Biotope type arable fields	H11	Extensive cropland use for field breeding birds and arable wild herbs
	H12	Set-aside of arable field with soil cover development on its own for species protection reasons; yield index < 2.500
	H13	Set-aside of arable field with soil cover development on its own for species protection reasons; yield index > 2.501 and < 3.500
	H14	Set-aside of arable field with soil cover development on its own for species protection reasons; yield index > 3.501
	H15	Set-aside with stubble (as single measure; compare W05)
Biotope type meadows	H20	Conversion of cropland into grassland
	H21	Extensive mowing of valuable habitats (from a nature conservation perspective); cutting date 01.06.
	H22/F22	Extensive mowing of valuable habitats (from a nature conservation perspective); cutting date 15.06.
	H23/F23	Extensive mowing of valuable habitats (from a nature conservation perspective); cutting date 01.07.
	H24/F24	Extensive mowing of valuable habitats (from a nature conservation perspective); cutting date 01.08.
	H25/F25	Extensive mowing of valuable habitats (from a nature conservation perspective); cutting date 01.09.
	H26/F26	Extensive mowing of valuable habitats (from a nature conservation perspective); cutting until 14.06., management stop between 15.06. and 31.08.
	H27	Abandonment of any kind of fertilizers and chemical pesticides (as single measure; compare N21)
	H28	Preservation of orchards (single fruit trees) (as single measure; compare W07)
	H29	Set-aside of meadows for species protection reasons
	H30	Result-oriented grassland use
Biotope type pastures	H31/F31	Extensive grazing of valuable habitats (from a nature conservation perspective); grazing of sheep, cattle incl. water buffalo, or horses incl. donkeys
	H32/F32	Extensive grazing of valuable habitats (from a nature conservation perspective); grazing of cattle on alps (alpine areas)
	H33/F33	Extensive grazing of valuable habitats (from a nature conservation perspective); grazing of goats
Biotope type ponds	H41	Support for ecologically valuable ponds with silting zone (*Verlandungszone*); alternative 1: < 25% silting zone
	H42	Support for ecologically valuable ponds with silting zone; alternative 1: > 25% silting zone
	H43	Support for ecologically valuable ponds with silting zone; alternative 2: < 25% silting zone
	H44	Support for ecologically valuable ponds with silting zone; alternative 2: > 25% silting zone
	H45	Complete non utilization of ponds
Additional services (for VNP measures)	N11	Abandonment of any kind of fertilizers
	N12	Abandonment of mineral fertilizers and organic fertilizers (except solid dung)
	N21	Abandonment of any kind of fertilizers and chemical pesticides
	N22	Abandonment of mineral fertilizers, organic fertilizers (except solid dung) and chemical pesticides
	W01	Reduced seed density
	W02	Long commute (minimum 5 km one-way)
	W03	Management unit (=field size) maximum 0.5 ha
	W04	Management unit (=field size) maximum 0.3 ha

	W05	Set-aside with stubble
	W06	Annual management procedure for soil cultivation between 01.09. and 31.10.
	W07	Preservation of orchards (single fruit trees)
	W08	Use of mowing device with cutter bars
	W09	Use of special machines
	W10	Use of motor mowers (*Motomäher*)
	W11	Mowing by hand
	W12	Scraping together by hand
	W13	From a nature conservation perspective required additional cut
	W14	Mandatory preservation of year-round old grass strips/areas on 5-20% of the area
	W15	Additional bonus for wet/litter meadows
	W16	Animal-friendly mowing
	W17	Stop of management from 15.03. or 01.04. until the fixed date
	W18	Accompanying goats
	W19	Management unit (=field size) maximum 2 ha
	W20	Draining of ponds (*Sömmerung*); maximum half of the pond is stocked (*bespannt*)
	W21	Stocking (*Bespannung*) from 01.03-15.09. and fast re-stocking
	U01	Abandonment of mechanical and thermal weed control
	U02	Pre-grazing (*Vorweide*) of the area prohibited until end of April
	U03	Early mowing strips or areas possible on maximum 20% of the area
	U04	Stop dry fishing in two of five years
	U05	Fishing with a fishing rod is not allowed
	U06	Dry fishing until 31.12.

B. Information for the sampling process

Table B1. Institutions contacted during the E-mail survey for the nature managers and additional remarks.

**Institutions**	**Additional remarks**
Upper nature conservation authority & lower nature conservation authorities	Each upper authority of the seven Bavarian districts (*Regierungsbezirke*) was contacted with the request to forward the survey to the respective subordinate lower authorities
National parks	Berchtesgaden and Bavarian Forest
Biosphere reserves	Rhön and Berchtesgadener Land
Nature parks	Information center Eichstätt (with the request to forward the survey to rangers and offices)
Area manager (*Gebietsbetreuer*):	Coordination office (with the request to forward the survey)
Wildlife habitat consultant (*Wildlebensraumberater*): -	Coordination office (with the request to forward the survey)
Nature protection guards (*Naturschutzwacht*):	Coordination office (with the request to forward the survey)
"AK Stadtgrün" (*Stadtgrün* = city green)	Survey was first sent to the Association of Bavarian Cities (*Bayerischer Städtetag*) with request to forward survey
Green agencies (*Grünämter*)	Survey was first sent to the Association of Bavarian Cities (*Bayerischer Städtetag*) with request to forward survey
Landscape architects	Bavarian coordination office of the Federation of German Landscape Architects (*Bund Deutscher Landschaftsarchitekten*) was point of contact
Ecostation Straß	In representation of the members of the Bavarian Academy for Nature Conservation and Landscape Management (*Bayerische Akademie für Naturschutz und Landschaftspflege*)
Associations for Landscape Management	State coordination of the Bavarian Association was contacted with the request to forward the survey
German Federation for the Environment and Nature Conservation	Big environmental association
State Association for Bird Protection (*Landesbund für Vogelschutz*)	Big environmental association
German Alpine Club (*deutscher Alpenverein (DAV)*)	Smaller association
Association for the protection of the mountain landscape (*Verein zum Schutz der Bergwelt e.V.*)	Smaller association
Association for landscape management and species protection (*Verein für Landschaftspflege und Artenschutz e.V.*)	Smaller association

Table B2. Counts of respondents working/engaging in the respective organization. Only respondents that answered the open-ended questions regarding improvements for subsidy possibilities were considered. Respondents could select multiple organizations; therefore, double counts exist.

**Organization**	**Type of engagement**	**Count**
National park	Employer nature	2
Biosphere reserve	Employer nature	0
Upper nature conservation authority	Employer nature	13
Lower nature conservation authority	Employer nature	18
District (*Landkreis*)	Employer nature	7
Area manager	Employer nature	20
Nature park	Employer nature	15
Associations for Landscape Management	Employer nature	11
Nature protection guards	Employer nature	13
Other (e.g., landscape architects and housing associations)	Employer nature	20
City administration	Employer urban	8
Municipal administration	Employer urban	3
Church community	Employer urban	0
Nature protection association	Honorary	31
Nature protection guards (for lower nature conservation authority)	Honorary	18
Municipality (maintenance of residential green)	Honorary	3
Church community	Honorary	1
Other (e.g., coordination center for bats)	Honorary	6

C. Description of the categorization system for suggestion according to their novelty or pre-existence

Table C1. Detailed description of the categories, including subcategories, for the differentiation of new and existing ideas, and the number of suggestions in each category for nature managers (nat), farmer cluster 1 (f1), and farmer cluster 2 (f2).

Codes	Description	nat	f1	f2
new and substantially different approaches for AES	All answers that suggested new ways of developing AES, including ideas for changing the administrative system, and answers that suggested new measures which are not supported yet by the AES or the LNPR; answers were also seen as new if other support programs outside the AES might exist but if alternative support through AES or LNPR was conceivable; answers were additionally included if they could have been seen as adaptation but made a very specific description and mentioned exact monetary values	40	123	87
comments & buzzwords regarding existing AES	All one-word answers or very short phrases or comments without suggestions for improvement that addressed topics which are already part of the AES and LNPR; as an example "late cutting date" was classified as buzzword, but "later cutting dates" was classified as adaptation as "later" suggests that the existing cutting dates are too early	44	296	224
suggested adaptations for existing AES (and administration)	All answers that suggested and improvement, a change, an adaptation or similar, related to existing measures, existing programs (AES and LNPR) or the administrative system and the set-up/orientation of AES; suggestions were only included if a negative effect could be largely excluded, otherwise they were not coded	81	261	157
general orientation	All answers that suggested an adaptation of the general system, particularly the administration of the programs and the organization; some suggestions included topics that are related to AES, but the rules stem partly from the EU CAP like the contract length; these were still included in AES because a certain decision freedom exists within the countries	43	113	69
overall	All answers that suggested an adaptation of the general system, particularly the administration of the programs and the organization, that do not specifically target one program, e.g., either multiple programs were mentioned in the same answer, or no program was mentioned	39	99	63
only KULAP	All answers that suggested an adaptation for the KULAP; ideas were only coded if they specifically included the term "KULAP" or "Cultural Landscape Program" or if there was a clear hint that the suggestion was only applicable in the KULAP, e.g., "3 ha rule"	1	10	3

only VNP	All answers that suggested an adaptation for the VNP; ideas were only coded if they specifically included the term “VNP” or “Nature Conservation Program” or if there was a clear hint that the suggestion was only applicable in the VNP	3	4	3
Cultural Landscape Program (KULAP)	All answers that were linked to existing measures only within the Cultural Landscape Program	14	106	48
climate protection	All answers that were linked to existing measures only within the pillar for climate protection; a link was established if answers mentioned the codes in this pillar like “B20” or if answers described one of the measures, topics, or procedures within this pillar	0	4	2
soil & water protection	All answers that were linked to existing measures only within the pillar for soil & water protection; a link was established if answers mentioned the codes in this pillar like “B30” or if answers described one of the measures, topics, or procedures within this pillar	1	43	8
biodiversity	All answers that were linked to existing measures only within the pillar for biodiversity; a link was established if answers mentioned the codes in this pillar like “B43”, “B48”, or if answers described one of the measures, topics, or procedures within this pillar	2	35	16
cultural landscape	All answers that were linked to existing measures only within the pillar for cultural landscape; a link was established if answers described one of the measures, topics, or procedures within this pillar like “8 € per tree”; codes were not mentioned	1	7	10
organic farming	All answers that were linked to organic farming, including terms like “organic”, “organic farming”, “organic agriculture”; apart from the mentioned terms, a link was established if answers mentioned the respective code “B10”	8	12	9
overlapping	All suggestions that were addressing multiple pillars at the same time within the KULAP or that were only an adaptation to the KULAP (and already included in the VNP) and therefore not yet included in any pillar like “cutting date 15.06. in the Cultural Landscape Program”	3	6	3
Nature Conservation Program (VNP)	All answers that were linked to existing measures only within the Nature Conservation Program	7	3	1
biotope type arable fields	All answers that were linked to existing measures for the biotope type arable fields; a link was established if answers described one of the measures, topics, or procedures for this biotope type	0	1	0

biotope type pastures	All answers that were linked to existing measures for the biotope type pastures; a link was established if answers described one of the measures, topics, or procedures for this biotope type; since measures for “pastures” also exist within the KULAP, answers were mostly classified under the code e*quivocal (KULAP & VNP)* as a clear linkage to the VNP only was missing in most cases	1	1	0
biotope type meadows	All answers that were linked to existing measures for the biotope type meadows; a link was established if answers described one of the measures, topics, or procedures for this biotope type; since “meadows” and “grassland” are often used replaceable and measures for “meadows” also exist within the KULAP, answers were mostly classified under the code e*quivocal (KULAP & VNP)* as a clear linkage to the VNP only was missing in most cases	3	1	0
biotope type ponds	All answers that were linked to existing measures for the biotope type ponds; a link was established if answers described one of the measures, topics, or procedures for this biotope type;	0	0	1
additional services	All answers that were linked to additional services that can be combined with measures of the VNP; as similar measures exist in both programs or described suggestions are also part of measures in the KULAP, e.g., “abandonment of fertilizers”, answers were mostly classified under the code e*quivocal (KULAP & VNP)* as a clear linkage to the VNP only was missing in most cases	1	1	0
overlapping	All suggestions that were addressing multiple pillars at the same time within the VNP or that were only an adaptation to the VNP (and already included in the KULAP) and therefore not yet included in any pillar	2	0	0
equivocal (KULAP & VNP)	All answers that were linked to measures, topics, or procedures relevant in both programs and where no clear link to a single program could be established, including terms like “cutting frequency”, “conversion of cropland into grassland”, “better support for the abandonment of pesticides”, etc.	12	39	33
combination possibilities	All answers that addressed adaptations, ideas, or comments regarding combination possibilities between and among programs, mostly between and within the KULAP, VNP and organic farming, particularly including terms like “combination possibility”, “better combine” or similar, combined with the names of the programs	2	11	10

Landschaftspflege- und Naturparkrichtlinien	All answers that were primarily linked to the goals of the LNPR and had a focus on species and biotopes or specific landscapes like bogs and that did not have a clear linkage to measures of the VNP; for some classified ideas, support programs might already exist, due to the high number of regional or local support programs for single species, but they could not all be identified in this thesis	6	4	8

D. Thematic categorization system with code frequencies for farmers and nature managers

Table D1. Detailed description of all thematic categories. These descriptions are equally applicable to the categorization system of the farmers and nature managers. Codes are indented according to their level (subcategories). Codes in level 4 marked with a star (*) did not exist in the categorization system of the nature managers and were summarized under the respective code in level 3, as absolute numbers were too low for a further division into the subcategories.

Code-Level 1	2	3	4	Comment
agriculture & landscape				All answers related to agricultural practices, landscape structures, and the management and protection of the environment
	landscape structure			All answers related to landscaping, landscape/structural elements, biotopes, and landscape conservation
		field margins		All answers related to field margins, including terms like "(forest) margins", "field margins" or similar. Margins around water bodies were classified under *water protection*
			buzzwords field margins*	All one-word answers/one phrase without additional information, primarily the words "field margins" or "forest field margins"
			comments/ suggestions for field margins*	All comments and (new) suggestions for adaptations related to field margins including terms like "fields margins" combined with terms like "protection", "maintenance", "preservation", "more", etc.
			establishment of field margins*	All answers related to the establishment of field margins, including terms like "field margins" combined with terms like "establish", "establishment", "construct", etc.
		hedges		All answers related to hedges, primarily including the word "hedges"
			buzzwords hedges*	All one-word answers/one phrase without additional information, primarily the word "hedges"
			comments/ suggestions for hedges*	All comments and (new) suggestions for adaptations related to hedges including terms like "hedges" combined with terms like "protection", "maintenance", "preservation", "removal", "more", etc.
			establishment of hedges*	All answers related to the establishment of hedges, including terms like "hedges" combined with terms like "establish", "establishment", "plant", etc.
		biotopes/ structural elements		All answers related to biotopes and structural elements including terms like "ponds", "dead wood (piles)", "stone walls", "field shrubs", "alleys", "wet areas", "nature zones", etc.; hedges and field margins were excluded from this category

		buzzwords biotopes/ structural elements*	All one-word answers/one phrase without additional information, like the words “biotopes”, “landscape elements”, “field shrubs”, etc.
		comments/ suggestions for biotopes/ structural elements*	All comments and (new) suggestions for adaptations related to biotopes and landscape elements including terms like “ponds”, “dead wood (piles)”, “stone walls”, “field shrubs”, “alleys”, “wet areas”, “nature zones”, etc., combined with terms like “support”, “allow”, “more”, etc.
		establishment of biotopes/ structural elements*	All answers related to the establishment of biotopes and landscape elements including terms like “ponds”, “dead wood (piles)”, “stone walls”, “field shrubs”, “alleys”, “wet areas”, “nature zones”, etc., combined with terms like “establish”, “establishment”, “plant”, etc.
	biotope networks		All answers related to biotope networks, primarily the word “biotope” combined with terms like “connect”, “network”, etc.
	landscape conservation		All answers related to landscape conservation, including terms like “(cultural) landscape preservation/conservation”, “renaturation”, “diverse landscape”, etc.
	small-scaled structures/ fields		All answers related to small fields and small structures, often combined with terms like “(more) support”, “preserve”, etc.
	wildflower areas		All answers related to wildflower areas and flower strips, primarily including the terms “wildflower areas”, “flower strips”
		buzzwords wildflower areas*	All one-word answers/one phrase without additional information, primarily the words “wildflower areas”, “flower strips”
		comments/ suggestions for wildflower areas*	All comments and (new) suggestions for adaptations related to wildflower areas and flower strips including terms like “wildflower areas”, “B48”, “flower strips”, “colorful fallow”, “wild herbs”, etc., combined with terms like “(more) support”, “network”, “flexibility”, etc.
		establishment of wildflower areas*	All answers related to the establishment of wildflower areas and flower strips, including terms like “wildflower areas”, “flower strips”, etc., combined with terms like “establish”, “sowing”, etc.
animal husbandry			All answers related to animal husbandry, including terms like “animal husbandry”, “livestock (unit)”, “grazing”, etc.

	extensive animal husbandry	All answers related to extensive animal husbandry, including terms like “livestock”, “animal”, “factory farming” (*Massentierhaltung*) combined with terms like “limit”, “limitation”, “maximum”, “extensive”, etc.; not to confuse with the code *linkage production-livestock area*
	animal feed	All answers related to animal feed, including terms like “feed”, “feed import”, “local legumes”, “local feed”, etc.
	animal welfare	All answers related to animal welfare, including terms like “animal welfare”, “appropriate”, etc., combined with “animals”, “livestock transport", etc.
	linkage production-livestock area	All answers related to the link between livestock units and production area including terms like “livestock unit per ha”, “livestock unit less than”, “area-linked livestock”; compared to the code *extensive animal husbandry* a particular focus was put on the occurrence of the word “livestock unit” (livestock unit is usually measured per hectare) combined with words like “area”, “ha” or a certain number indicating the maximum livestock units
	support for grazing	All answers related to the support for grazing, primarily including the term “grazing” combined with “(more) support”; those segments were not included under the code *(agricultural) policy*
	grazing	All answers related to grazing, primarily including terms like “grazing”, “grazing systems”, “grazing animals”, etc.
	other suggestions for animal husbandry	All answers that mentioned “animal husbandry” or terms related to animal husbandry, but did not fit in any of the other categories
grassland		All answers related to grassland, including terms like “grassland”, “meadows”, etc.
	flower meadows	All answers related to flower meadows, including the term “flower meadows”; in contrast to wildflower area, a particular focus was put on the word “meadow”
	demand/support for grassland	All answers related to the demand for grassland, including one-word answers with “grassland” or combined with “preservation”, and also the (financial) support, including words like “grassland”, etc., combined with “more”, “support”, etc.; segments with support were not included under the code *policies & design*
	extensive grassland	All answers related to extensive grassland, primarily the term “grassland” combined with terms like “extensive”, “less fertilization”, “without fertilization”, etc.

	biodiverse meadows/pastures	All answers related to biodiverse meadows or pastures, primarily including the terms “meadows” and “pastures” combined with “biodiversity” or “biodiverse”; not to confuse with the code *flower meadows* where a particular focus was put on the occurrence of the term “flower meadows” itself
	cutting frequency	All answers related to the cutting frequency of grassland or meadows, including terms like “cutting frequency”, “mowing frequency”, “maximum cuts”, “cutting intensity”, etc., sometimes combined with “grassland” or similar
	cutting date	All answers related to cutting date of grassland or meadows, including terms like “cutting date”, “mowing date”, “late cut”, “later cut”, “maximum cuts”, etc., sometimes combined with “grassland” or similar
	animal-/insect-friendly mowing	All answers related to mowing practices that are beneficial for insects and animals, including terms like “animal friendly”, “insect friendly”, “mulching”, “double cutter bar”, etc.
	other suggestions for grassland	All answers that mentioned “grassland”, “meadows” or related terms, but did not fit in any of the other categories
	design of mowing practices	All answers related to the topic how mowing can be performed, including terms like “rotating mowed areas”
arable land		All answers related to arable land/cropland, especially farming practices, including a variety of terms explained in the respective categories
	cultivation of mixed crops/ perennials	All answers related to the cultivation of mixed crops including terms like “mixed crops on one field” and perennial crops, including the term “perennials”
	holistic/innovative agricultural systems	All answers related to agricultural systems that incorporate innovative ideas including a more holistic thinking, e.g., terms like “permaculture”, “three field crop rotation”, “agroforestry”, etc.
	reduction of mono-cultures & corn cultivation	All answers related to the reduction of monocultures and the cultivation of corn as an example for a monoculture, including terms like “monocultures” or “corn” combined with terms like “not any”, “limit”, “maximum”, “less”, etc.
	undersown crops	All answers related to undersown crops, primarily the word “undersown crops”

orchards		All answers related to orchards, including terms like “fruit trees”, “orchards”, etc., combined with terms like “support”, “more”, “maintenance”, etc.
fallow of arable land		All answers related to fallow of arable land, including terms like “set-aside (areas)”, “fallow”, “not cultivate”, etc.
crop rotation		All answers related to crop rotation, including terms like “diverse crop rotation”, “multiple crop rotation”
	buzzwords crop rotation*	All one-word answers/one phrase without additional information, like “diverse crop rotation”, “multiple crop rotation”, “wide crop rotation”, “changing crop rotation”, etc.
	comments/ suggestions for crop rotation*	All comments and (new) suggestions for adaptations related to crop rotation including terms like “diverse crop rotation”, “multiple crop rotation”, etc., combined with terms like “support”, “extend”, etc.
soil protective measures (no-till/ reduced till)		All answers related to soil protective measures, particularly including terms like “no-till” or “reduced till”, sometimes combined with the term “soil cultivation”; not to confuse with the category for *soil protection*
catch crops		All answers related to catch crops, including terms like “catch crops”, “winter soil cover”, “winter catch crops”, etc.
	Buzzwords catch crops*	All one-word answers/one phrase without additional information, primarily the terms “catch crops”, “winter soil cover”, “winter catch crops”
	comments/ suggestions for catch crops*	All comments and (new) suggestions for adaptations related to catch crops including terms like “catch crops”, “winter soil cover”, “winter catch crops”, etc., combined with terms like “support”, “extend”, “cultivate”, etc.
humus/soil fertility		All answers related to humus and soil fertility, including the terms “humus” or “soil fertility”
	buzzwords humus formation*	All one-word answers/one phrase without additional information, primarily the word “humus formation”
	comments/ suggestion for humus/soil fertility*	All comments and (new) suggestions for adaptations related to humus and soil fertility, including the terms “humus (formation)” and “soil fertility” combined with terms like “support”, “improvement”, “preservation”, etc.
fertilizer		All answers related to fertilizers, including terms like “fertilizer”, “manure”, “dung”, “nitrogen”, “ammonium”, etc.

	other comments/ suggestions*	All comments and (new) suggestions for adaptations related to fertilizers, that did not fit in any of the other subcategories for fertilizers
	methods for fertilizer reduction*	All answers related to the reduction of fertilizer, including terms like “fertilizer”, “manure”, “dung” combined with terms like “reduction”, “lower application”, “less”, “limitation”, “maximum”, “efficient”, etc.
	ban/abandonment manure only*	All answers with the terms “manure” or “dung” combined with “ban”, “abandonment” or “not any”
	ban/abandonment fertilizers*	All answers with the terms “fertilizer”, “mineral fertilizer”, “artificial fertilizer” or similar combined with “ban”, “abandonment” or “not any”
pesticides		All answers related to pesticides, herbicides, and fungicides, including terms like “pesticides”, “herbicides”, “fungicides”, “(chemical) plant protection”, etc.
	other comments/ suggestions*	All comments and (new) suggestions for adaptations related to pesticides, that did not fit in any of the other subcategories for pesticides
	methods for pesticide reduction*	All answers related to the reduction of pesticides including terms like “pesticides”, “herbicides” combined with terms like “minimal application”, “limit application”, “less”, etc.; also terms like “mechanical weed control”, “beneficials”, etc.
	ban/abandonment pesticides*	All answers with the terms “pesticides”, “spraying” or similar combined with “ban”, “abandonment” or “not any”
future-oriented agricultural practices/ crops		All answers related to practices or crops that address future considerations, including terms like “water efficient crops”, “drones”, “non-mutated crops”, “disease-resistant”, “climate resistant”, etc.
legumes/ protein crops		All answers related to legumes and protein crops, including terms like “legumes”, “protein crops”, “clover”, “lucerne”, etc.
sowing practices		All answers related to sowing practices, including terms like “mulch seeding”, “direct seeding”, “strip seeding”, “seeding intensity”, etc.
conversion cropland - grassland (both ways)		All answers related to the conversion of cropland into (permanent) grassland and grassland into cropland, including terms like, “grassland conversion”, “cropland in grassland”, etc.
status of agricultural fields		All answers related to the status of agricultural fields, especially in relations to the conversion rules after five years, including terms like “agricultural status”, “conversion”, combined with “remove 5-years rule”, “no conversion”, etc.

	ideas for different crops		All answers related to ideas for different crops that do not particularly address future issues e.g., "traditional crops", "hemp"
environment			All answers related to the environment and environmental goods, including terms like “biodiversity”, “nature”, “water”, “soil”, etc., often combined with terms like “protection”, “important”, “support”, etc.; if idea was not addressing environmental good directly, related topics must have been specifically mentioned to be included in the category for environment, e.g., mulch seeding is beneficial for soil protection but was only included in the respective category if the respondent mentioned both terms (“mulch seeding” and “soil protection”) together
	soil protection		All answers related to soil protection, including terms like “soil protection” or “soil conservation”; not to confuse with the code *soil protective measures*, focusing on “no-till” or “reduced till”
		land consolidation	All answers related to land consolidation, particularly including the term “land consolidation”
		soil protection in general*	All answers related to soil protection including suggestions that specifically mentioned terms like “soil protection”, “soil conservation”, “soil improvement”, etc., or answers including terms like “lighter machines”, “exhaustion of soils” or similar that were not addressing erosion protection
		erosion protection*	All answers related to erosion protection, particularly including the term “erosion” combined with terms like “protection”, “strips”, etc.
	CO2 & climate		All answers related to CO2 and climate, including terms like “climate”, “carbon (storage)”, “CO2”, “dryness”, “etc.
		management regarding water resources*	All answers related to the management of water resources, especially answers regarding rainfed agriculture, including terms like “limit water use”, “water-efficient”, “water storage”, etc.
		climate protection/ adaptation*	All answers related to climate protection and climate adaptation, e.g., practices that addressed climate protection or answers including terms like “climate change” or similar
		CO2 sequestration*	All answers related to the sequestration of CO2, including terms like “sequestration”, “store”, etc.; ideas were only included if CO2 sequestration or the connection between sequestration and climate was expressed in the answers, e.g., “humus formation” was not included but “humus formation and sequestration of carbon” was included
	water protection		All answers related to water protection, particularly including the terms “(ground) water protection”, “riparian strips”, etc.

	buzzwords water protection	All one-word answers/one phrase without additional information, primarily the word “(ground) water protection”
	riparian strips*	All answers related to riparian strips; ideas were only coded if there was a clear relation, e.g., through terms like “riparian”, “bank”, etc., combined with terms like “strip” or similar
	suggestions for water protection*	All comments and (new) suggestions for adaptations related to water protection in general; ideas were only coded if answers included specifically terms like “water protection”, “water bodies”, etc., combined with “protection” or similar or had a clear linkage, including terms like “extensification of wet areas” or similar
biodiversity		All answers related to biodiversity, particularly including terms like “biodiversity”, “biodiverse”, “species-rich”, etc.; not to confuse with species protection summarized under the codes for *environmental/nature/species protection*
	Comments/ suggestions for biodiversity*	All one-word answers/one phrase without additional information, primarily the words “biodiversity”, “biodiverse”, “species diversity” or similar
	buzzwords biodiversity*	All comments and (new) suggestions for adaptations related to biodiversity, including terms like “biodiverse”, “biodiversity”, “species diversity”, etc., combined with terms like “grassland”, “support”, “keep”, etc.
environmental/ nature/species protection		All answers related to environmental, nature and/or species protection; ideas were only coded if answers specifically included the terms “environmental”, “species”, “nature” or related terms combined with terms like “protection”, “importance”, etc.
	bees*	All answers related to bees; ideas were only included if answers specifically included the terms “bees” or “apiculture”
	other (forest) animals	All answers related to (forest) animals; ideas were only coded if answers specifically included terms like “wildlife”, “beavers”, “rabbits”, “wolves”, etc., combined with “protection”, “quiet zones”, etc.; answers including the word “hamster” were coded in a separate category as some answers addressed specific hamster programs
	hamsters*	All answers related to hamsters; ideas were only coded if answers included the term “hamster”
	birds	All answers related to birds; ideas were only coded if the answers included terms like “birds”, “bird species”, “ground-breeding birds”, etc., or specific bird species

			insects	All answers related to insects; ideas were only coded if answers included the term “insects” or specific insect species, e.g., answers addressing mulching were only included if “mulching” and terms related to “insects” were mentioned together
			general (environmental/ nature/species protection)	All answers related to environmental, nature and species protection, that addressed the topic in more general terms and not one group of species in particular, including terms like “species protection”, “(pristine) nature”, “nature protection”, etc.
		ecosystem		All answers related to the ecosystem as a whole, particularly including the term “ecosystem”
research, consulting & education				All answers related to research, consulting and education, including terms like “education”, “information”, “consultants”, “consulting”, “research”, etc., combined with terms like “society”, “children”, “farmers”, etc.
	(environmental) education of society			All answers related to the (environmental) education of the society, including terms like “education”, “information”, etc., combined with terms like “population”, “society”, “humans”, “children”, “school”, “relations”, etc.
		society		All answers related to (environmental) education of the society, without answers that included terms particularly linked to children like “children”, “pupils”, “schools”, “teens”
		children		All answers related to (environmental) education, particularly of children; answers were only coded if they included the terms “children”, “pupils”, “schools”, “teens”
	education/ training for farmers			All answers related to education and training of farmers, including terms like “education”, “information”, “consultants”, consulting”, “research”, etc., combined with term like “farmers” or terms related to farms like “education independent of production system”, etc.
	advisory/ support services			All answers related to advisory support and other support services, particularly including terms like “advice” and “support”, often combined with terms like “for farms”, “for farmers”, “regarding measures”, etc.; support in this case was not understood as monetary support
		general*		All answers related to advisory support and other support services in general, including terms like “information” or “consultancy”, etc., combined with terms like “farm”, “farmers”, “farm system”, etc.; without answers that included terms like “individual”, etc.

		for individual farms*	All answers related to individual advisory support and individual support services, particularly including terms like “individual”, “personal”, “on-site”, etc., combined with terms like” information”, “consultancy”, “concepts”, etc.
	research/ alternative methods		All answers related to research and out-of-the-box thinking beyond the pure agricultural system, including terms like “research”, “solar and crops”, etc.
policies & design			All answers related to policies, politics, and the design of support programs with relation to AES, including regulations, policies and subsidies/subsidy programs with a variety of suggestions and terms explained in the respective categories
	importance of regionality		All answers related to the importance of regional production, regional products, and feed imports, including terms like “production in Bavaria”, “self produced feed”, “production for own use”, “regionality”, “regional programs”, “less foreign feed”, etc.
	public/ municipal authorities/ politics		All answers related to public and municipal authorities and politics; ideas were particularly coded when they included terms like “municipality”, “authority”, “AELF” or similar
	reduction in bureaucratic/ administrative efforts		All answers related to the reduction of bureaucracy and the reduction of administrative efforts regarding measures and programs, including terms like “bureaucracy”, “administration”, “effort”, etc., combined with terms like “less complex”, “reduction”, “less”, “complicated”, etc.
	flexibility/ practicability/regionality of programs		All answers related to the wish for more flexible and practicable measures and a better regional adaptation, including terms like “flexibility”, “practicability”, “easier”, “less complicated”, “more regional”, “adapted to regional conditions”, etc., combined with terms like “programs”, “measures”, “KULAP”, “VNP” or specific measures, etc.
	demand for stricter regulations		All answers related to the demand for stricter regulations; ideas were particularly coded when they included terms like “control”, “check”, “ban”, “strict”, “mandatory”, “law” or similar
	desire for autonomy/ exchange		All answers related to the desire for autonomy and exchange (particularly between farmers and the society), including terms like “voluntary”, “exchange”, “let farmer work”, “reduce influence of NGOs”, “decision-making freedom for farmers”, “talk together”, “include experiences”, etc.
	criticism of existing regulations		All answers related to criticism of existing regulations and programs; ideas were only coded if signs for criticism could be observed in the wording, including exclamation marks, irony, or terms like “exploited”, “incomprehensible”, “(not) meaningful”, “abolish”, “absurd” or similar

future orientation of the agricultural policy	All answers related to the future orientation and direction of the agricultural policy; this category is very diverse and summarizes also topics that were related to the agricultural policy but where no other category was suitable; ideas that were coded addressed the EU CAP, topics outside the agricultural sphere like “support for diversification”, topics related to a new way of thinking like “support for quality instead of quantity” or included terms like “aim”, “direction”, “until now”, “abolish”, “overview”, “create”, etc.
price policy of agricultural products	All answers related to the price policy of agricultural products and ideas that criticize the cheap prices, including terms like “better/higher/fairer prices”, “adequate prices system”, “prices stability”, etc.
(regional) marketing of agricultural products	All answers related to the (regional) marketing of agricultural products; ideas were only coded if they addressed specifically marketing, including terms like “better marketing possibilities”, “support for direct marketing”, “regional marketing”, etc.; if answers only addressed regionality they were coded as *importance of regionality*
organic farming as solution	All answers related to organic farming, particularly including terms like “organic”, “organic farming”, “organic agriculture” or similar; ideas including terms related to organic that were combined with terms like “support” or similar were coded *as support for organic farming*
extensification as solution	All answers related to extensification, including terms like “extensification”, “extensive”, “less intensive”, “lower intensity”, etc., combined with terms like “agriculture”, “area”, “management”, etc.; ideas for extensive grassland were not included here but coded as *extensive grassland*
biogas production	All answers related to the production of biogas and biogas substrate, including terms like “biogas”, “energy crops”, “corn”, etc., combined with terms like “limit”, “not any”, “reduction”, “wildflower areas”, etc.
reinforcement of individuality	All answers related to ideas that expressed the wish to reinforce more individuality, including terms like “own ideas”, “individual seed mixtures”, “farm specific measures”, etc.; if ideas included terms related to advisory services it was also coded thereunder as *individual farms*
planning and legal certainty	All answers related to planning and legal certainty with regard to programs, regulations, and monetary support, including terms like “long-term”, “longer-term”, “more binding”, “continuity”, “earlier announcement”, etc., combined with terms like “support”, “planning”, “measures”, etc.

financial support & support programs			All answers related to financial support and support programs in a more narrow sense, e.g., flexibility of programs is also related to support programs however this category focuses more directly on ideas for mainly financial support and specific programs like the AES, including a variety of terms explained in the respective categories
	design of AES		All answers related to the design of AES in a narrwo sense, with respect to time frame, area setting and area dependent regulations
		time frame*	All answers related to the design of AES in a narrow sense, with respect to time frame, area setting and area dependent regulations, including terms like “area-dependent”, “time frame”, “area setting”, etc., often combined with terms related to measures or the AES in general
		area setting*	All answers related to the time frame within AES, including terms like “time frame”, “year to year”, etc.
		area dependent regulations*	All answers related to the area setting within AES, including terms like “area setting”, “all areas”, “easier accessibility”, etc., combined with terms like “adaptation”, “extension”, etc.
		future-oriented design of AES*	All answers suggesting ideas for the restructuring of AES not related to time, frame, area setting or area-dependency
	Cultural Landscape Program (KULAP)		All answers related to the Kulturlandschaftsprogramm; ideas were only coded if measures within the Kulturlandschaftsprogramm were specifically addressed, including (the codes of) measures like “B43” or if answers included the terms “KULAP” or “Kulturlandschaftsprogramm”
	Nature Conservation Program (VNP)		All answers related to the Vertragsnaturschutzprogramm; ideas were only coded if measures within the Vertragsnaturschutzprogramm were specifically addressed, including (the codes of) measures like “H11” or if answer included the terms “VNP” or “Vertragsnaturschutzprogramm”
	Greening		All answers related to the Greening; ideas were only coded if answers included the term “Greening” or related terms like “ecological focus areas”
	support for organic farming		All answers related to the (financial) support of organic farming, including terms like “organic”, “organic agriculture”, etc., combined with terms like “support”, “money”, “bonus”, etc.; answers were either coded in this category if they included “support” or related terms or they were coded as *organic farming as solution* (no double coding between the two codes)

combination possibilities between programs	All answers related to the possibility of combining different programs and combining measures within programs, including term like “combination possibilities”, “combine”, etc., combined with terms like “programs”, “measures”, “KULAP”, “VNP”, “organic farming”, etc.
(higher) support dependent on farm size	All answers related to (higher) support dependent on farm size, particularly including ideas with (higher) support for small farms
result-based (support) systems	All answers related to support based on success and performance, including ideas for various criteria without profit/monetary orientation, including terms like “success-oriented”, “success-based”, “according to biodiversity”, “coupled to environmental protection”, etc., often combined with terms like “support”, “subsidies”, etc.
demand for higher financial support	All answers related to the demand expressed for higher financial support; financial support was seen in a narrow sense, which means that ideas were only coded when there was a clear connection to the financial or monetary side, expressed in terms like “more money”, “financial improvement”, “higher bonuses”, “better remuneration”, etc.; contrary “more support” was not included because it could also imply advisory support
support for nature sustaining practices	All answers related to (agricultural) practices with the idea of sustaining/ improving the natural environment; this category comprises (farming) practices particularly in combination with the word “support”, like “support for catch crops”, “support for orchards”, “support for water protection”, etc., with the aim to stress the importance of/the need for support expressed in the answers
support for different aspects	All answers related to the support of different aspects; similarly to the code *support for nature sustaining practices,* this category comprises different goods, actions, criteria that are not directly practices in a narrow sense, particularly in combination with terms like “support”, “bonus” or similar, e.g., “scrapping bonus for pigsties”, “financial incentive for rural area”; the category does only include ideas that were not already coded in other categories for support like *support for grazing* and that were not already coded in a category within the code *(agricultural) policy*, to avoid unnecessary doubling
support for small-scaled structures/fields	All answers related to the support for small fields and small-scaled structures, including terms like “small areas”, “small fields”, “small structures”, etc., combined with the term “support” or related terms; all answers that were coded as *small-scaled*

	structures/fields and similar ideas that additionally included terms related to "(monetary) support" are in this category
support for future-oriented agricultural practices/ crops	All answers related to the support for future-oriented agricultural practices/crops, including terms like "climate-resilient seeds", "new technologies", etc., combined with the term "support" or related terms; all answers that were coded as *future-oriented agricultural practices/crops* and similar ideas that additionally included terms like "(monetary) support" are in this category

Table D2. Thematic categorization system with frequencies of coded segments. Indent indicates the respective level in the code system. For nature managers, crossd out categories did not exist in the categorization system as absolute numbers of suggestions were too low for further division.

Codes	farmer cluster f1	farmer cluster f2	nature managers
agriculture & landscape	521	355	101
landscape structure	137	111	23
field margins	16	9	5
buzzwords field margins	7	0	-
comments/suggestions for field margins	6	6	-
establishment of field margins	3	3	-
hedges	24	23	5
buzzwords hedges	4	4	-
comments/suggestions for hedges	10	7	-
establishment of hedges	10	12	-
biotopes/structural elements	20	37	7
buzzwords biotopes/structural elements	3	10	-
comments/suggestions for biotopes/structural elements	11	11	-
establishment of biotopes/structural elements	6	16	-
biotope networks	4	3	3
landscape conservation	6	6	4
small-scaled structures/fields	9	11	2
wildflower areas	64	26	2
buzzwords wildflower areas	28	11	-
comments/suggestions for wildflower areas	33	11	-
establishment of wildflower areas	3	4	-
animal husbandry	30	20	12
extensive animal husbandry	0	1	0
animal feed	3	2	1

animal welfare	0	1	2
linkage production-livestock area	4	1	1
support for grazing	5	4	6
grazing	15	6	3
other suggestions for animal husbandry	4	7	0
grassland	77	49	21
flower meadows	9	4	1
demand/support for grassland	8	7	5
extensive grassland	12	6	0
biodiverse meadows/pastures	6	3	2
cutting frequency	6	8	2
cutting date	20	12	8
animal-/insect-friendly mowing	6	3	3
other suggestions for grassland	5	5	3
design of mowing practices	13	6	0
arable land	246	139	26
cultivation of mixed crops/perennials	7	6	1
holistic/innovative agricultural systems	5	10	1
reduction of monocultures/corn cultivation	5	6	1
undersown crops	5	1	1
orchards	11	18	0
fallow of arable land	21	11	5
crop rotation	40	12	1
buzzwords crop rotation	14	5	-
comments/suggestions for crop rotation	26	7	-
soil protective measures (no-till/reduced till)	10	1	1
catch crops	36	9	1

buzzwords catch crops	12	1	-
comments/suggestions for catch crops	24	8	-
humus/soil fertility	15	9	2
buzzwords humus formation	5	5	-
comments/suggestion for humus/soil fertility	10	4	-
fertilizers	34	30	7
other comments/suggestions for fertilizers	3	4	-
methods for fertilizer reduction	20	15	-
ban/abandonment manure only	1	4	-
ban/abandonment fertilizer	14	10	-
pesticides	26	19	4
other comments/suggestions for pesticides	4	3	-
methods for pesticide reduction	18	11	-
ban/abandonment pesticides	5	8	-
future-oriented agricultural practices/crops	13	8	1
legumes/protein crops	9	4	0
sowing practices	14	2	0
conversion cropland - grassland (both ways)	8	5	0
status of agricultural fields	1	1	0
ideas for different crops	9	7	0
environment	99	85	45
soil protection	19	14	0
land consolidation	0	0	-
soil protection in general	9	9	-
erosion protection	10	6	-
CO2 & climate	14	7	4
management regarding water resources	3	0	-

climate protection/adaptation	5	5	-
CO2 sequestration	6	2	-
water protection	22	22	3
riparian strips	12	3	-
buzzwords water protection	2	8	-
comments/suggestions for water protection	8	11	-
biodiversity	19	16	13
buzzwords biodiversity	7	4	-
comments/suggestions for biodiversity	12	12	-
environmental/nature/species protection	33	37	29
bees	3	2	0
other (forest) animals	8	11	1
hamsters	0	1	0
birds	9	9	6
insects	5	6	1
general (environmental/nature/species protection)	14	15	23
ecosystem	4	1	
research, consulting & education	9	18	10
(environmental) education of society	0	0	0
society	0	0	-
children	0	0	-
education/training for farmers	1	7	1
advisory/support services	8	10	9
general	2	6	-
for individual farms	6	4	-
research/alternative methods	0	1	0
policies & design	314	209	111

importance of regionality	1	0	7
public/municipal authorities/politics	4	2	0
reduction in bureaucratic/administrative efforts	14	14	6
flexibility/practicability/regionality of programs	44	8	11
demand for stricter regulations	1	4	3
desire for autonomy/exchange	14	1	1
criticism of existing regulations	8	4	0
future orientation of the agricultural policy	0	0	0
price policy of agricultural products	1	0	0
(regional) marketing of agricultural products	2	0	0
organic farming as solution	9	18	2
extensification as solution	14	5	5
biogas production	1	0	0
reinforcement of individuality	4	4	1
planning/legal certainty	5	7	2
financial support & support programs	226	161	84
design of AES	41	29	11
time frame	2	3	-
area setting	4	6	-
area dependent regulations	15	12	-
future-oriented design of AES	20	10	-
Cultural Landscape Program (KULAP)	39	21	1
Nature Conservation Program (VNP)	14	14	11
Greening	2	0	0
support for organic farming	11	11	8
combination possibilities between programs	12	11	2
(higher) support dependent on farm size	3	3	2

result-based (support) systems	5	9	15
demand for higher financial support	29	18	10
support for nature sustaining practices	71	44	20
support for different aspects	9	9	8
support for small-scaled structures/fields	4	6	2
support for future-oriented agricultural practices/crops	2	5	2

E. Exemplary coding of two suggestions

Example 1, “higher support for the cultivation of catch crops, instead of glyphosate application”, was coded as follows:

Table E1. Exemplary coding for the suggestion of example 1.

Codes			
all usable suggestions			
	suggested adaptations for existing AES (and administration)		
		Kulturlandschaftsprogramm	
			soil & water protection
agriculture & landscape			
	arable land		
		catch crops	
			comments/suggestions for catch crops
		pesticides	
			methods for pesticide reduction
(agricultural) policy			
	financial support & support programs		
		demand for higher financial support	

Example 2, “support wildflower areas or similar on approx. 20% of the farm area, so that it can be integrated into the farm business as element of the crop rotation and design the remuneration in such a way that one is financially better off than if one cultivates corn or similar”, was coded as follows:

Table E2. Exemplary coding for the suggestion of example 2.

Codes			
all usable suggestions			
	suggested adaptations for existing AES (and administration)		
		Kulturlandschaftsprogramm	
			biodiversity
agriculture & landscape			
	landscape structure		
		wildflower areas	
			comments/suggestions for wildflower areas
	arable land		
		reduction of monocultures/corn cultivation	
		crop rotation	
			comments/suggestions for crop rotation
(agricultural) policy			
	financial support & support programs		
		support for nature sustaining practices	

F. Specific farmer suggestions and comparison

Specific new suggestions per cluster:

Examples for aspects only mentioned in f1 were the cultivation of corn only once in five years, and the inclusion of 30% permanent grassland into the crop rotation. Another idea was to improve payments for grassland for a better compensation compared to cropland, with a payment of 500 € for a maximum of three cuts. One respondent suggested to establish new agricultural branches like “landscape conservation” with mostly extensive cultivation. Further proposals included the cultivation of various crops (water efficient, disease resistant) and bonuses , for suckler cow husbandry, or for wild birds breeding on the farm. In cluster f2, ideas were (the support for) perennial crops, permaculture, agriphotovoltaics, as well as the inclusion of weather adapted crops. Moreover, a bonus for animal husbandry without manure and the for set-aside of agricultural land for very long periods, like 15 years, were mentioned. Two very specific suggestions were a “three-part KULAP for catch crops: Stage 1: after the harvest of the grains, grass-clover mix is mown in autumn and fed and directly turned over (umbrechen): bonus 75 €/ha. [Stage 2:] clover is mown and fed in autumn. Afterwards turn over and subsequent crop: 150 €/ha. Stage 3: winter soil cover without the use as fodder as usual: 300 €/ha” as well as a “subsidy for the following type of grassland management: No artificial fertilizer (especially nitrogen) only dung and liquid manure on the meadow of the own cows. Maximum number of animals per hectare area (lease and ownership) No calf rearing with anything else other than the milk from the mother cow and green fodder from the own land. No use of protein crops/feed (soy from South America), not tethering; open stable and grazing.”.

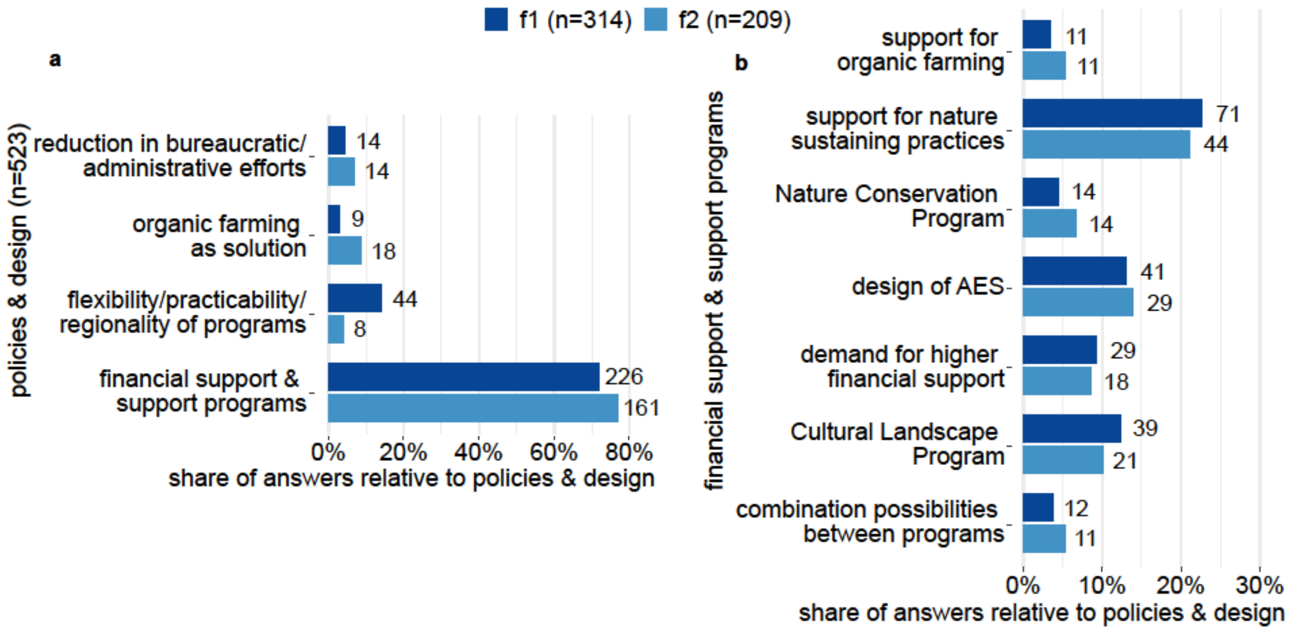

Fig. F1. Relative share of the most dominant codes within (a) policies & design and (b) financial support & support programs as a subcode of policies & design for the group of farmers per cluster. Numbers behind the bars indicate absolute numbers (counts) of coded segments. Codes are displayed if the respective code a share of at least 5% in at least one cluster, relative to the counts for policies & design.

Adaptations:

Table F1. Detailed description of selected farmer suggestions

	Existing measures	Adaptations	Cluster
**KULAP**		Climate protection	
	B19, B20, (B21) (extensive grassland use; gazing stock eating roughage)	- More varieties and intensities for LSU<br>- B20: only consider LSU that really need roughage	f1<br>f1
	B25/B26 (low emission distribution of organic fertilizers)	- Extend fertilizer application close to ground	f1
	B28/B29* (conversion crop-grassland along water bodies)	- Allow conversion into grassland in all areas<br>- Remove limit of 5 ha; e.g., allow on 0,1 % of the cropland in upper Bavaria	both<br>f2
		Soil & water protection	
	B32/B33, B34* (riparian and erosion protection strips)	- Compensation according to soil quality<br>- Active support for wide riparian strips (>10m)	f1<br>f1
	B35* (winter soil cover with catch crops)	- Re-establish/support measure for catch crops/undersown crops<br>- Extend catch crop cultivation<br>- Without fixed date for soil cultivation<br>- Support for the cultivation of catch crops without specific crops<br>- Enable support for catch crops for small farms without greening	both<br>f1<br>f1<br>f1<br>f1
	B37 (mulch seeding)	- Re-establish/support mulch seeding<br>- Improved practicability, e.g., if mulch cover can't be established due to dryness	both<br>f1
		Biodiversity	
	B40 (preservation of biodiverse grasslands)	- Include cutting date<br>- Include alternating mowing practices (between hay and silage)	f1<br>both

	B43-B46* (diverse crop rotation)	- Extend crop rotation (program)<br>- Stop B43 because supports conventional rape cultivation<br>- Include fallow in crop rotation (with remuneration)<br>- (More support for) the cultivation of (domestic) legumes, alfalfa, clover; include alfalfa grass (f2); also for organic farms	f1<br>f1<br>f1<br>both
	B47 (yearly changing wildflower areas), B48/B61* (wildflower areas)	- Enable stepwise mulching for patches with old and new plants<br>- Support interconnected wildflower strips<br>- Extend/remove area limitations; e.g., allow wildflower areas on 20% of farm area (enables option for inclusion into crop rotation), or on entire farm area, or for 20 ha of farm area<br>- Implement sowing period late in the year/after harvest to establish a plant stock (*Bestand*) before winter, that stays until the harvest in the consecutive year; no turn over before April<br>- more flexibility, e.g., regarding deviations of only 20% (f4)<br>- Support wild herbs instead of flowering strips<br>- Individual seed mixtures	f1<br>f1<br>both<br>f1<br>f1<br>f2<br>f2
	B49 (renewal of hedges/field shrubs) including investment measure	- More support for the maintenance of hedges<br>- Improved protection of hedges<br>- Inclusion of field margins; maintenance and more protection	f1<br>f2<br>f2
		Cultural landscape	
	B57* (orchards)	- Protection of fruit tress without size restrictions	f2
	B59* (structural and landscape elements) including investment measure	- Higher remuneration for planting trees at field margins<br>- Possibility to remove landscape elements (ahead of fixed timeframe)<br>- Inclusion of further landscape elements: stone houses, dead wood structures, clearance cairns, stone piles<br>- More biodiverse and blooming hedges<br>- Allow more landscape elements per area	f1<br>f1<br>both<br>f2<br>both
		Organic farming	
	B10* (organic farming)	- Remove minimum LSU of 0.3; the main forage area should not be considered	f2
		Overlapping	
	Applicable to several measures	- Attractive support for measures with very late mowing only every two years (good for ground-breeding birds and plant diversity)<br>- Reduced seed density (now only in VNP)<br>- Set-aside for longer/temporary periods (mostly in VNP, in KULAP not subsidized)<br>- More possibilities for extensive grassland	f1<br>f1<br>both<br>f1
**VNP**		Biotope type arable fields/meadows	
	H12-H14 (fallow arable field), H29 (fallow meadow)	- Adapt measures to avoid mulching every two years on fallow areas	f1
		Biotope type ponds	
	H41-44	- Less support for ponds free of fish, and more for managed fishponds (kingfisher density increases with intensive K0-breeding)	f2

G. Specific nature managers' suggestions

Table G1. Suggested adaptations by nature managers for the KULAP and the VNP. Codes and measures were extracted from the overview of measures of the KUALP and the VNP (version December 2020, (StMELF, 2020b)).

	Existing measures	Adaptations
KULAP		Soil & water protection
	B35 (winter soil cover with catch crops)	- Re-establish undersown crops (to support undersown crops in corn)
		Biodiversity
	B49 (renewal of hedges/field shrubs) including investment measure	- More effort to protect hedges<br>- Inclusion of alleys
		Cultural landscape
	B59 (structural and landscape elements) including investment measure	- Inclusion of alleys
		Overlapping
	Applicable to several measures	- Include nature-preserving techniques, e.g., cutter bars (now only in VNP)<br>- Re-establish set-aside; set-aside of 7-10% of the farm area (now mostly in VNP, in KULAP mostly not subsidized)
VNP		Biotope type pastures
	H31-H33, F31-F33 (extensive grazing)	- Improved financial support, especially for alpine regions (see example: Switzerland)
		Biotope type meadows
	H21-H26, F22-F26 (extensive mowing)<br>all meadow measures	- Flexibility for cutting dates (yearly variations: dryness, precipitation, etc.)<br>- strong use of old grass strips in all meadow related measures
		Additional services
	W08	- improved support for double cutter bars

H. Comparison between suggested measures and AES participation data

*Table H1. The top five measures regarding the **area enrolled** and **number of applications** for the KULAP and the VNP for the year 2020. Changes in the position with respect to area enrolled/number of applications are shown for the years 2018 and 2019.For area, the dataset contains only area values for measures offered in 2015 (StMELF, 2014; Appendix A). For number of applications, all measures from the leaflet for 2021 are considered (StMELF, 2020b; Appendix A, C). Source: (European Commission, 2014a, 2014b)both provided by the StMELF.*

Position 2020/ 19/18	KULAP - Code & measure description		Position 2020/ 19/18	VNP - Code & measure description	
**Enrolled area**					
1/1/1	B26/ B25	Low emission distribution of organic fertilizers	1/1/1	N21	Abandonment of any kind of fertilizers and chemical pesticides
2/2/2	B10	Organic farming	2/2/2	H22	Extensive mowing; cutting date 15.06.
3/4/3	B44	Crop rotation with legumes	3/3/3	W17	Management stop until fixed cutting date
4/3/5	B45	Crop rotation with large grain legumes	4/4/4	H23	Extensive mowing; cutting date 01.07.
5/5/4	B20	Extensive grassland use for gazing stock eating roughage, maximum 1.4 livestock units	5/5/5	W15	Additional bonus for wet/litter meadows
**Number of applications**					
**1/1/2**	B26/ B25	Low emission distribution of organic fertilizers	1/1/1	N21	Abandonment of any kind of fertilizers and chemical pesticides
**2/3/3**	B60	Summer grazing (bonus)	2/2/2	H22	Extensive mowing; cutting date 15.06.
**3/2/1**	B30	Extensive grassland use along water bodies and in sensitive areas	3/3/3	H23	Extensive mowing; cutting date 01.07.
**4/4/5**	B10	Organic farming	4/4/4	W15	Additional bonus for wet/litter meadows
**5/6/7**	B48	Wildflower areas at the forest edge and in the field	5/5/5	W17	Management stop until fixed cutting date

I. Survey items and modified variables for the cluster analysis

Table I1. Variables with respective questions from the LandKlif survey used for the cluster analysis of the farmers. Table indicates where new variables were established based on questionnaire items or where items had to be transformed for the analysis.

<table>
<tr><th>Variable name</th><th>Original question</th><th>Response possibilities</th></tr>
<tr><td>act_spo,
act_wild,
act_ntfp,
act_mot,
act_fihu,
act_oth,
act_non,
count_sports</td><td>For which private activities do you use nature and landscapes in Bavaria? Please select (multiple answers possible)</td><td>- walking, hiking, running, or biking
- watching wildlife
- collecting mushrooms, wild herbs, or berries
- trips by motorcycle or car
- fishing or hunting
- other (please indicate)
- no private activities</td></tr>
<tr><td colspan="3">Modification: For count_sports, all sport activities that were selected were summed up resulting in a maximum value of 6 and a minimum value of 0 (= no private activity).</td></tr>
<tr><td>term_ES</td><td>Have you ever heard the term ecosystem services?</td><td>yes
no</td></tr>
<tr><td>LU_ES</td><td>For what should nature and landscape be used in Bavaria?
Please distribute points according to the importance. – Preserve services of nature and landscape for humans.</td><td>scale from 0 to 100</td></tr>
<tr><td>LU_bdiv</td><td>For what should nature and landscape be used in Bavaria?
Please distribute points according to the importance. – Support biodiversity of plants, animals, and mushrooms.</td><td>scale from 0 to 100</td></tr>
<tr><td>LU_inc</td><td>For what should nature and landscape be used in Bavaria?
Please distribute points according to the importance. – Gain financial income from forestry and agricultural products.</td><td>scale from 0 to 100</td></tr>
<tr><td>relation</td><td>For a successful protection of nature and landscape, are especially important for Bavarian agricultural landscapes…
… personal relation of farmers to nature</td><td>Likert scale with the possibilities:
- very unimportant
- unimportant
- neither
- important
- very important
- I am not sure</td></tr>
<tr><td colspan="3">Modification: The binomial variable relation_unsure was derived from this question. For this, all respondents that indicated “I am not sure” received a “yes” while respondents that did not indicate anything received a “no”. The same procedure was applied for the variables laws_unsure (based on laws), submeas_unsure (based on submeas) and suboutc_unsure (based on suboutc).</td></tr>
<tr><td>laws</td><td>For a successful protection of nature and landscape, are especially important for Bavarian agricultural landscapes…
… stricter legal regulations</td><td>Likert scale with the possibilities:
- very unimportant
- unimportant
- neither
- important
- very important
- I am not sure</td></tr>
</table>

submeas	For a successful protection of nature and landscape, are especially important for Bavarian agricultural landscapes…<br>… subsidies for prescribed measures	Likert scale with the possibilities:<br>- very unimportant<br>- unimportant<br>- neither<br>- important<br>- very important<br>- I am not sure
suboutc	For a successful protection of nature and landscape, are especially important for Bavarian agricultural landscapes…<br>…subsidies based on success	Likert scale with the possibilities:<br>- very unimportant<br>- unimportant<br>- neither<br>- important<br>- very important<br>- I am not sure
particip_AES	Are you participating in agri-environmental schemes at the moment (KULAP, VNP, LNPR)?	yes<br>no
climcha_crop,<br>climcha_grass	Do you already notice any changes as a result of climate change on your own cultivated areas?	- yes, on cropland<br>- yes, in grassland<br>- no
**Modification:** Question was split into two variables, one for grassland and one for cropland, with each having "yes" or "no" as response possibility.		
occ_status	Which connection do you have to farming?	- owner of a farm business<br>- family member or employee working in the farm business<br>- other
operation_mode	Farming is for you?	- full-time<br>- part-time
farm_sys	What type of farming do you do?	- conventional<br>- organic
area_crop,<br>area_grass,<br>area_forest	What is the size of your cultivated area (rounded up, in hectare)	Blank boxes to write down number for the following possibilities:<br>Survey 1:<br>- cropland<br>- permanent grassland<br>- other agricultural area (e.g., viticulture, fruit production (*Obstbau*)<br>- forest<br>- sum (of all)<br><br>Survey 2:<br>- cropland<br>- extensive permanent grassland – cut (incl. partly grazing)<br>- extensive permanent grassland – grazing only<br>- intensive permanent grassland<br>- forest<br>- sum (of all)

<table>
<tr><td colspan="3">Modification: This question had different answer possibilities as survey 2 gathered additional information for another research project.
For homogenization, the variables were constructed as follows:
area_crop: the area of cropland (equal in both survey)
area_grass: for survey 1, the area of permanent grassland was taken; for survey 2 the sum of all three grassland options was taken
area_forest: the area of forest (equal in both surveys)</td></tr>
<tr><td>anim_onsite</td><td>Do you have animals in the following animal groups (including young animals (Jungtiere))? Please, to the extent known, indicate in livestock units (LSU).</td><td>Blank boxes to write down number for the following possibilities:
Livestock units (if known):
- cattle
- …of those dairy cattle
- pigs
- poultry
- sheep
- goats
- horses, donkey, mule (Maultier)

Number of animals (if LSU not known)
- cattle
- …of those dairy cattle
- pigs
- poultry
- sheep
- goats
- horses, donkey, mule</td></tr>
<tr><td colspan="3">Modification: This question was transformed into a binomial variable indicating whether at least one animal is present on the farm. Every respondent that wrote down a value >0 in at least one of the 14 boxes related to the 14 animal categories received a “yes”, the rest received a “no”.</td></tr>
<tr><td>age*</td><td>What age category do you belong to?</td><td><18, 18-25, 26-30, 31-35, 36-40, 41-45, 46-50, 51-55, 56-60, 61-65, 65-70, >70</td></tr>
<tr><td colspan="3">Modification: Age categories were summarized and transformed into an ordinal variable with three classes:
young: <18, 18-25, 26-30, 31-35
middle: 36-40, 41-45, 46-50, 51-55
old: 56-60, 61-65, 65-70, >70</td></tr>
<tr><td>sex*</td><td>What is your gender?</td><td>- female
- male
- divers
- no answer</td></tr>
<tr><td colspan="3">Modification: No answer was considered as missing value.</td></tr>
</table>

educ	What general school education do you have? Please indicate only the highest one.	- lower secondary school<br>- middle secondary school<br>- higher secondary school<br>- polytechnic secondary school<br>- without general education degree<br>- no answer

Modification: Polytechnical secondary school (only selected four times in the entire sample) was classified as middle secondary school (both finish in 10th grade). For variables in cluster analysis naming changed:

lower secondary school (*Haupt-(Volks-)Schulabschluss*)→ basic_school
middle secondary school (*Realschul- oder gleichwertiger Abschluss*) + Polytechnic secondary school (*polytechnischen Oberschule*)→ medium_school
higher secondary school (*Fachhochschul- oder Hochschulreife*)→ high_school
without general education degree → no_school
No answer was considered as missing value.

This variable was defined as ordinal with the order: no_school < basic_school < medium_school < high_school

quali	My professional training is (multiple answers possible):	- no agricultural training<br>- professional school<br>- apprenticeship<br>- agricultural school<br>- master diploma<br>- academy<br>- college<br>- university

Modification: Qualifications were summarized and transformed into an ordinal variable with four classes (based on the German qualification framework (*deutscher Qualifikationsrahmen*):
non: no agricultural training
basic: professional school (*Berufsschule/Berufsfachschule (ohne betriebliche Lehre)*), apprenticeship (*Berufsausbildung/Lehre (Gehilfen-, Facharbeiter- oder andere Abschlussprüfung)*)
intermediate: agricultural school (*Landwirtschaftsschule (auch Weinbau-, Gartenbau-, Winterschule)*), master diploma *(Meister, Fachagrarwirt)*, academy (*Höhere Landbauschule, Technikerschule, Fachakademie*), college (*Fachhoch-, Ingenieurschule*)
advanced: university (*Universität, Hochschule*)

climcha_atti	How do you feel about the following statements? Select.<br>Climate change in general…<br>- …is scientifically verifiable (1)<br>- …has predominantly natural causes (2)<br>- …has already global impacts today (3)<br>- …will only become relevant for future generations (4)<br>- …can still be influenced (5)	- no, for sure not<br>- no, probably not<br>- I am not sure<br>- yes, probably<br>- yes, for sure

Modifications: The questions were summarized and transformed into a variable representing the attitude towards climate change. Each respondent was shown a random selection of three questions out of five. The question whether climate change can still be influenced, was not considered since this depends strongly on a very personal view and can scientifically neither be rejected nor accepted.

For the other four questions a scheme was developed, and the following values were attributed to each respondent:
belief: respondents that answered with "yes, …" to question 1, 3 and with "no, …" to question 2, 4

insecure belief: respondents that answered with “yes, …” or “I am not sure” to question 1, 3 and with “no, …” or “I am not sure” to question 2, 4
insecure: respondents that answered all questions with “I am not sure”
insecure rejection: respondents that answered with “no, …” or “I am not sure” to question 1, 3 and with “yes, …” or “I am not sure” to question 2, 4
rejection: respondents that answered with “no, …” to question 1, 3 and with “yes, …” to question 2, 4
indifferent: respondents that had different combinations where no trend was observed

Indifferent and insecure were in the end treated as one class, namely insecure.

J. Additional information for the farmers' cluster analysis

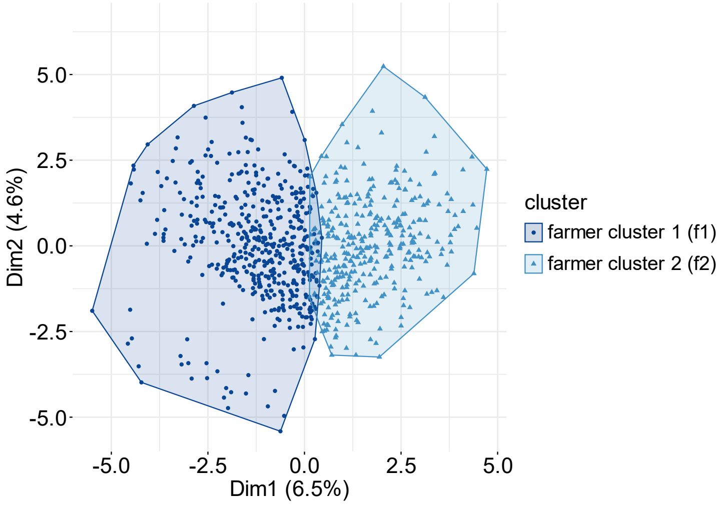

Fig. J1. Farmer clusters as a result of the hierarchical clustering and the NbClust function.

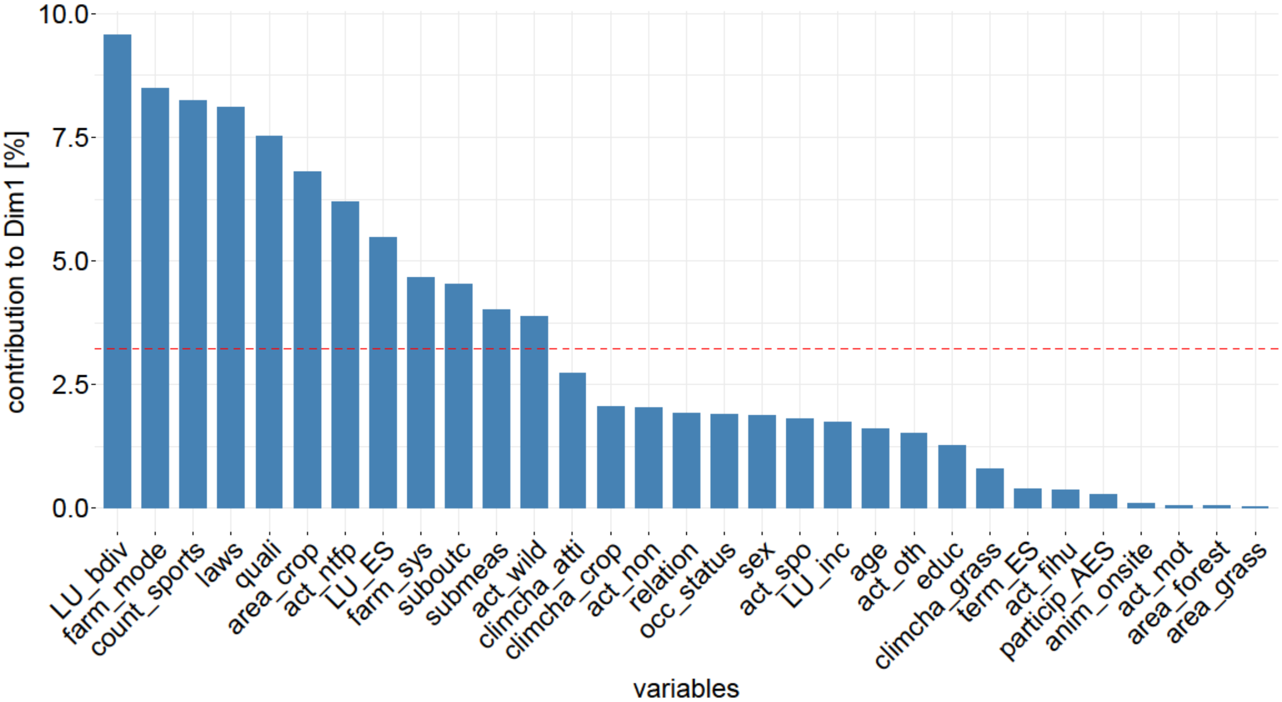

Fig. J2. Contribution of each variable to dimension one. Values represent with how many percent one variable contributes to the construction of dimension one. If a variable has a high contribution, it means that the respective dimension explains a lot of the variance in the data with respect to this variable.

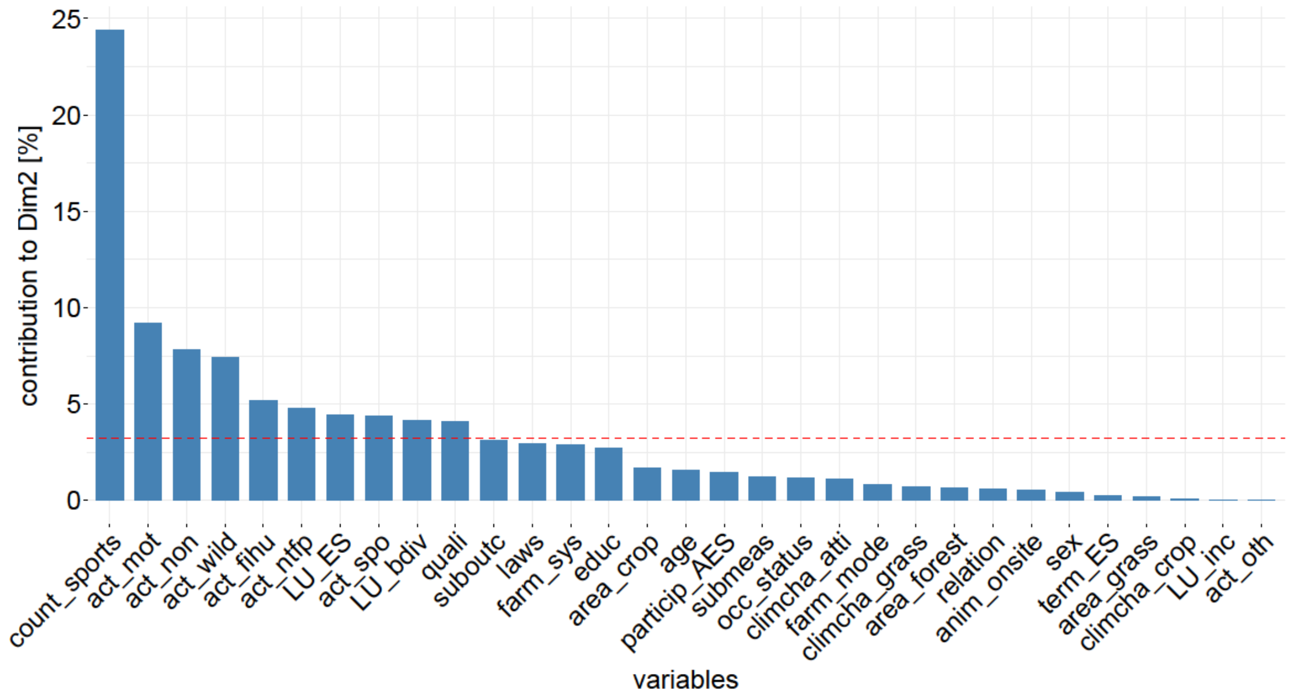

Fig. J3. Contribution of each variable to dimension two (farmers). Values represent with how many percent one variable contributes to the construction of dimension two. If a variable has a high contribution, it means that the respective dimension explains a lot of the variance in the data with respect to this variable.

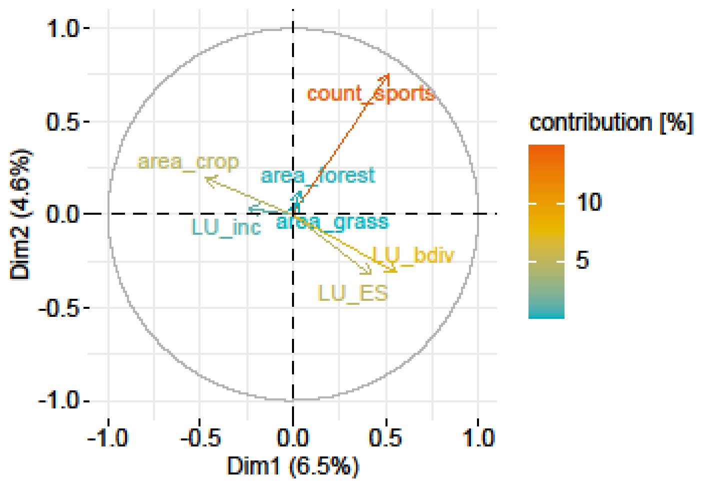

Fig. J4. Contribution (strength and direction) of the quantitative variables to the first two dimensions. Dark orange colors have *a high contribution to the dimensions. For variable names, see SI, Table I1.*

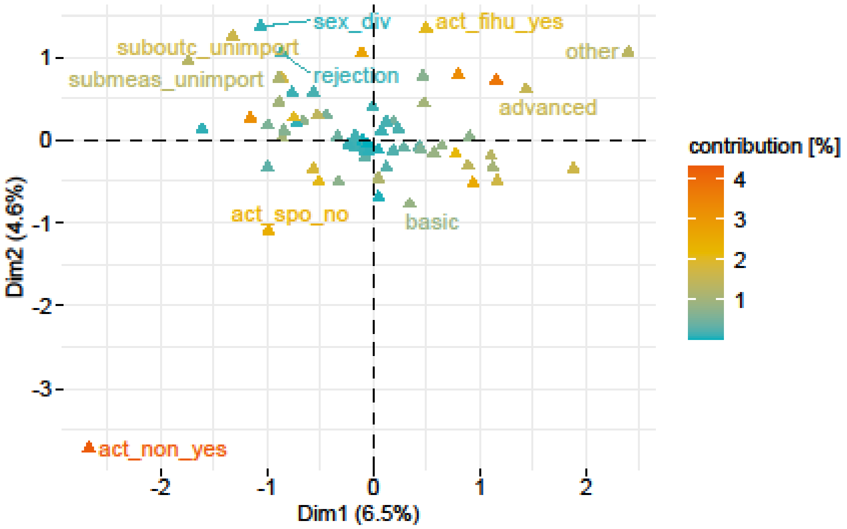

Fig. J5. Contribution (strength and direction) of the qualitative variables to the first two dimensions. Dark orange colors have a high contribution to the dimensions. Axis values represent the correlation between the variable and the dimension. A stronger correlation implies a higher explanatory value with regard to the respective dimension. Variables around the center (0.0, 0.0) have a very low contribution and are not included with names in this Figure. Their contribution can be inferred from Fig. A4 and A5. For variable names, see SI, Table I1.

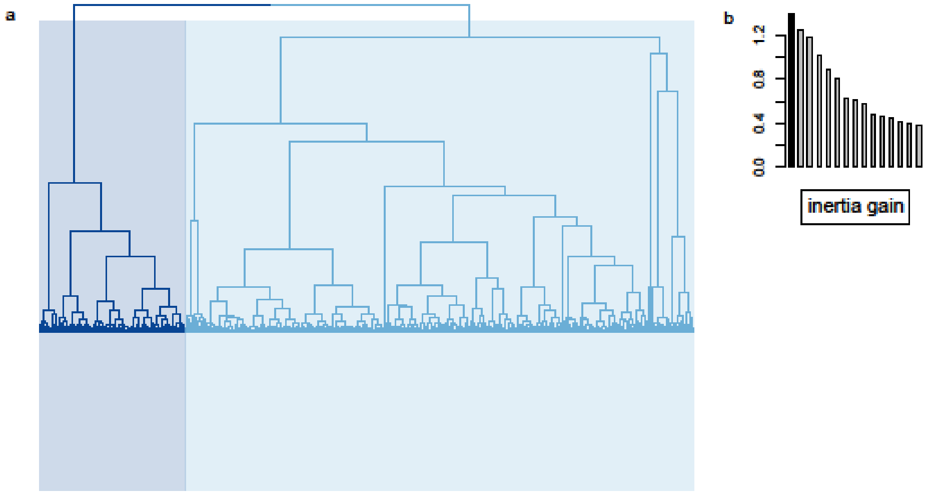

Fig. J6. (a) Dendrogram computed by the HCPC function. A total of 20 dimensions with an eigenvalue bigger than one were extracted with an explained variance of 58.31%. The hierarchical clustering yielded an optimal number of three clusters with multiple overlaps, and a possible division into two clusters, seen above. From the NbClust function most indices supported the finding of four clusters, followed by two clusters, as shown in the SI, Fig. J7. Based on this, the group of farmers were divided into two clusters (b) Bar plot with inertia gain computed by the HCPC function, showing the division into the two farmer clusters. The best partition is the one with higher relative loss in inertia (drop between neighbouring bars). The bar plot shows that the loss in inertia is also high between cluster two and three as well as three and four (bar one and tow as well as two and three, respectively). However, cluster results with three or four clusters had many overlaps/very small cluster sizes and two clusters were well supported by the NbClust function (see SI, Fig J7).

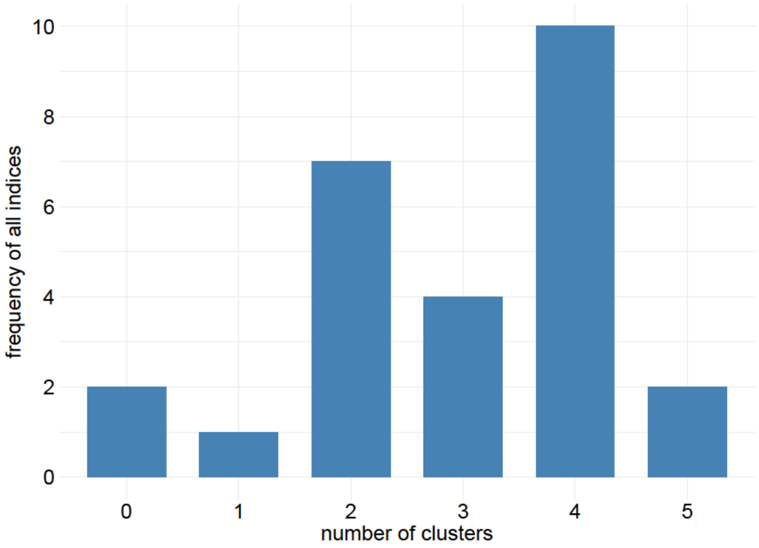

Fig. J7. Optimal number of clusters according to the NbClust function. The number of clusters with the highest frequency of indices is seen as optimal. The minimum and maximum number of clusters was set to two and five, respectively. We decided to divide the farmers into two cluster as a division into four cluster resulted in many overlaps among the clusters and one of the four clusters contained only three participants.

K. Description of the cluster characteristics for the farmers

Table K1. The ten most influencing qualitative variables for each farmer cluster. The clusters are described on the basis of the statistical v-test (describing the deviation between the mean of a subpopulation and the general average (Husson et al., 2020)) and their equivalent p-values. A p-value of 0.05 equals a v-test smaller/greater than -1.96/1.96 (Husson et al., 2010). Positive v-test values indicate that the average of the variable in the cluster is larger than the average of the variable in the sample. This means that if a v-test is higher than 1.96, the mean value of a specific category in one cluster is significantly higher than the overall mean in the sample. Exemplary, cluster f1 shows a p-values of 5.02e-24 and a v-test value of 10.11 for the category farm_sys = conventional. This indicates that there are significantly more farmers in f1, that work on a conventional farm (74.58% of farmers in f1), compared to the overall share of conventional farmers in the entire sample (60.00% of all farmers). Only the variables with ten most significant positive v-test values (significance was based on the v-test and the p-values) are listed and only the p-values are reported in the Table. SX. The design of the table was based on Beltrán-Alcrudo et al. (2018).

Qualitative variable name	Variable outcome	% of sample in the cluster [1]	% of variable outcome in the cluster [2]	% of variable outcome in the sample [3]	p-value
Cluster f1					
operation_mode	full-time	82.22	53.96	38.18	1.14e-29
quali	intermediate	76.27	68.96	52.61	3.94e-29
act_ntfp	no	71.24	80.00	65.33	1.50e-25
farm_sys	conventional	72.32	74.58	60.00	5.20e-24
act_wild	no	70.08	71.25	59.15	7.47e-17
laws	unimport	79.70	32.70	23.88	5.14e-13
laws	very unimportant	78.26	26.25	19.52	3.38e-09
relation	important	74.77	33.33	25.94	6.09e-09
suboutc	neither	80.30	25.01	16.00	6.30e-09
climcha_crop	yes	65.80	68.13	60.24	5.33e-08
Cluster f2					
act_ntfp	yes	69.78	54.20	32.48	1.12e-29
operation_mode	part-time	57.02	76.52	56.12	2.53e-24
act_wild	yes	61.44	56.81	38.67	1.15e-19
farm_sys	organic	63.74	50.43	33.10	3.40e-19
quali	advanced	79.27	18.84	9.94	4.16e-13
laws	important	68.75	28.70	17.45	7.97e-13
laws	very_important	83.87	15.07	7.52	2.05e-12
quali	non	68.12	27.25	16.73	9.69e-12
suboutc	very_important	61.01	38.55	26.42	2.91e-11
submeas	very_important	54.78	56.52	43.15	5.12e-11

1. Percentage of sample in the cluster = % of individuals with the respective variable outcome in the entire sample, that belong to this cluster. For example, 72.32% of all conventional farmers in the sample belong to cluster f1.

2. Percentage of variable outcome in the cluster = % of individuals in the cluster with variable outcome. For example, 74.58% of the farmers in cluster f1 are conventional.

3. Percentage of variable outcome in the sample = % of the respective variable outcome in the entire sample. For example, 60.00% of farmers in the entire sample are conventional which means that the rest is either organic or gave no answer.

Table K2. Socio-demographic and farm characteristics per each farmer cluster. Numbers do not add up to 100% as missing values are not shown.

Variable	Variable outcome	Absolute numbers in the cluster		Percent values in the cluster [numbers in %]	
		f1	f2	f1	f2
Farm system	conventional	358	252	75	44
	organic	99	234	21	41
Operation mode	full-time	259	98	54	17
	part-time	199	398	41	70
Participation in AES	yes	434	461	90	81
	no	45	82	9	14
Age	< 18	0	0	0	0
	18-25	18	2	4	1
	26-30	28	10	6	3
	31-35	32	14	7	4
	36-40	45	25	10	8
	41-45	48	32	11	10
	46-50	76	48	17	15
	51-55	90	59	20	18
	56-60	54	62	12	19
	61-65	44	48	10	15
	66-70	16	14	4	4
	> 70	5	8	1	2
Gender	male	403	364	84	64
	female	29	104	6	18
	divers	3	3	0	1
Education	no_school	0	1	0	0
	basic_school	149	138	31	24
	medium_school	173	130	36	23
	high_school	126	218	26	38
Qualification	non	44	140	1	25
	basic	64	96	13	17
	intermediate	331	169	69	30
	advanced	17	88	35	15
